# Mutation of p53 increases the competitive ability of pluripotent stem cells

**DOI:** 10.1242/dev.202503

**Published:** 2024-01-19

**Authors:** Salvador Perez Montero, Pranab K. Paul, Aida di Gregorio, Sarah Bowling, Solomon Shepherd, Nadia J. Fernandes, Ana Lima, Rubén Pérez-Carrasco, Tristan A. Rodriguez

**Affiliations:** ^1^ National Heart and Lung Institute, Imperial College London, Hammersmith Hospital Campus, Du Cane Road, London W12 0NN, UK; ^2^ Department of Life Sciences, Imperial College London, South Kensington Campus, London SW7 2AZ, UK; ^3^ Imperial BRC Genomics Facility, Imperial College London, Hammersmith Hospital Campus, Du Cane Road, London W12 0NN, UK

**Keywords:** Cell competition, p53, Mathematical modelling

## Abstract

During development, the rate of tissue growth is determined by the relative balance of cell division and cell death. Cell competition is a fitness quality-control mechanism that contributes to this balance by eliminating viable cells that are less fit than their neighbours. The mutations that confer cells with a competitive advantage and the dynamics of the interactions between winner and loser cells are not well understood. Here, we show that embryonic cells lacking the tumour suppressor *p53* are ‘super-competitors’ that eliminate their wild-type neighbours through the direct induction of apoptosis. This elimination is context dependent, as it does not occur when cells are pluripotent and it is triggered by the onset of differentiation. Furthermore, by combining mathematical modelling and cell-based assays we show that the elimination of wild-type cells is not through competition for space or nutrients, but instead is mediated by short-range interactions that are dependent on the local cell neighbourhood. This highlights the importance of the local cell neighbourhood and the competitive interactions within this neighbourhood for the regulation of proliferation during early embryonic development.

## Teaser

**Summary:** Pluripotent cells with p53 mutations gain a competitive growth advantage via the apoptotic elimination of wild-type cells.

## INTRODUCTION

The regulation of growth and tissue homeostasis during embryogenesis relies on the proper balance of cell division and cell death. One mechanism contributing to this balance is cell competition. Cell competition is a quality-control mechanism conserved from *Drosophila* to mammals that eliminates cells that, although viable, are less fit than their neighbours. During competition, those cells that are eliminated are generically termed losers. Accompanying this elimination, the fitter cells (winners) undergo compensatory proliferation, maintaining tissue homeostasis (reviewed by Baker, 2020; Bowling et al., 2019; Díaz-Díaz and Torres, 2019; Johnston, 2014; Madan et al., 2018; Maruyama and Fujita, 2017; Vishwakarma and Piddini, 2020). For the purpose of this study, we define cell fitness as the ability of a cell to thrive in its environment. This ability is likely to be determined by a number of parameters, including signalling ability, metabolic rates and cell adhesion properties, but ultimately is established by the balance between the cell division rate and the sensitivity to cell death.

An important implication of cell competition is that cellular fitness is not only a cell-intrinsic property, but is also determined relative to the fitness of neighbouring cells – a cell that is of sub-optimal fitness in one context may be ‘super-fit’ in the context of a different cell population. This is most clearly demonstrated in the case of wild-type cells in *Drosophila* and in mammals that can eliminate a range of defective cells (reviewed by Bowling et al., 2019), but can in turn also be eliminated by cells that over-express *Myc* (Clavería et al., 2013; de la Cova et al., 2004; Moreno and Basler, 2004; Sancho et al., 2013; Villa Del Campo et al., 2014). This ability to induce the elimination of wild-type cells has led to cells over-expressing *Myc* being termed ‘super-competitors’. The observation that cell fitness is relative to the cells’ neighbours implies that cells can interpret their relative fitness levels during cell competition. However, little is known regarding the mechanisms by which this process takes place. In *Drosophila* and in cancer cells, differential expression of *Flower* isoforms can act as fitness fingerprints for the winner and loser status of cells (Madan et al., 2019; Rhiner et al., 2010). Similarly, in *Drosophila* innate immune-like signalling has been shown to induce the elimination of *Minute* cells (which have a ribosomal deficiency) when they are surrounded by wild-type cells and the elimination of wild-type cells by *Myc* super-competitors (Meyer et al., 2014). However, this pathway is not required for competition when the flies are maintained in a sterile environment (Germani et al., 2018) and promotes the overgrowth of polarity-deficient cells when they are surrounded by wild-type cells (Katsukawa et al., 2018).

In mouse, at the onset of embryonic differentiation cell competition has been demonstrated to mediate the elimination of defective cells (Sancho et al., 2013) as well as those cells with low levels of *Myc* expression (Clavería et al., 2013). In the post-implantation embryo, just prior to gastrulation, this process eliminates around 35% of embryonic cells as a result of repression of the mTOR pathway (Bowling et al., 2018). This large-scale elimination is thought to ensure that only the fittest cells go on to contribute to further development and the germline (Bowling et al., 2019), and one important trigger of this elimination is mitochondrial dysfunction and mitochondrial DNA mutations (Lima et al., 2021). In the pre-implantation mouse embryo, signalling via the Hippo pathway is required for elimination through cell competition of mis-patterned cells (Hashimoto and Sasaki, 2019) and this pathway also mediates the elimination of normal human embryonic stem cells (ESCs) by karyotypically abnormal ones (Price et al., 2021), explaining how the abnormal cells expand in human pluripotent stem cell cultures.

Importantly, the cells eliminated in the early post-implantation mouse embryo display elevated P53 (TRP53) levels (Bowling et al., 2018; Lima et al., 2021) and cells with increased P53 expression are also eliminated during mouse during organogenesis (Zhang et al., 2017). In Madin–Darby canine kidney (MDCK) cells, mutation of the cell polarity gene *Scribble* in a mosaic fashion leads to an increase in P53 that induces mutant cell elimination via mechanical cell competition (Wagstaff et al., 2016). The observation that in mouse chimeras *p53* mutant cells have a selective advantage during development (Dejosez et al., 2013) and in the pluripotent state (Valverde-Lopez et al., 2023 preprint) suggests that loss of P53 expression provides cells with a competitive advantage. A further fascinating indication of the role of P53 in competition comes from analysing interspecies chimeras as well as co-cultured human and mouse pluripotent stem cells. Both *in vivo* and *in vitro*, human ESCs are eliminated by mouse ESCs by cell competition. However, mutation of *p53* in the human cells is sufficient to prevent the elimination of human ESCs (Zheng et al., 2021). Collectively, these data suggest that relative P53 levels are a key determinant of embryonic fitness. In this study, we explored the potential for p53 mutations to transform mouse cells into super-competitors. Through a comparative analysis of wild-type and p53 mutant cells in both separate culture and co-culture settings, and by employing a mathematical model to describe this dynamic, our findings reveal that p53-null mutant mouse ESCs actively trigger the apoptotic elimination of wild-type cells when co-cultured. Furthermore, we also find that this elimination is mediated by short-range signalling, highlighting the importance of local competitive interactions for the regulation of cell proliferation during the onset of differentiation.

## RESULTS

### P53 mutant ESCs behave as super-competitors

**Fig. 1.**
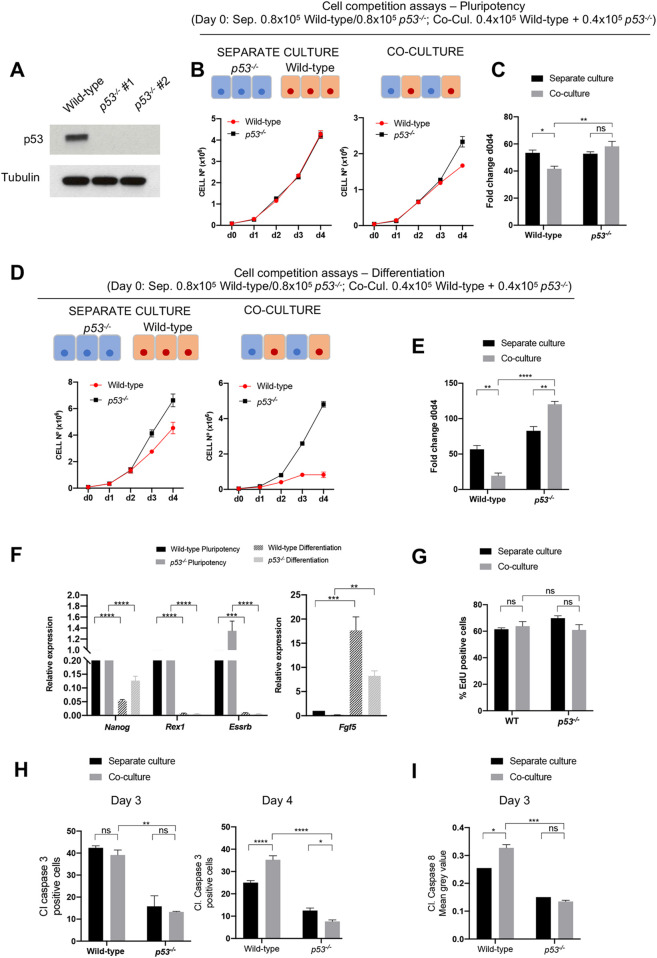
**Wild-type cells are outcompeted by *p53^−/−^* cells.** (A) p53 levels in wild type and two different *p53^−/−^* clones untreated or treated with the p53 activator Nutlin-3a for 4 h. (B) Growth curves of wild-type and *p53^−/−^* cells over 4 days in separate culture or co-culture (as indicated by schematics) in pluripotency conditions. (C) Fold change in wild-type and *p53^−/−^* cell numbers between day 0 and day 4 when cultured alone or co-cultured in pluripotency conditions. (D) Growth curves of wild-type and *p53^−/−^* cells over 4 days in separate culture or co-culture differentiation conditions. (E) Fold change in wild-type and *p53^−/−^* cell numbers between day 0 and day 4 when cultured alone or co-cultured in differentiation conditions. (F) Quantitative RT-PCR showing gene expression levels of naïve and primed pluripotency markers in wild-type and *p53^−/−^* ESCs in pluripotency and differentiation. Gene expression is normalised to beta-actin. (G) Percentage of EdU incorporation in wild-type cells and *p53^−/−^* cells cultured for 4 days separately or co-cultured. (H) Percentage of cleaved (Cl) caspase 3 positive cells determined by flow cytometry in wild-type and *p53^−/−^* cells cultured for 3 and 4 days separately or co-cultured. (I) Levels of cleaved caspase 8 in wild-type and *p53^−/−^* cells cultured alone or as co-culture for 3 days. Data were obtained from three independent experiments and are shown as mean+s.e.m. *P<0.05, **P<0.01, ***P<0.001, ****P<0.0001. d, days; ns, not significant.

The observation that in the embryo and in ESC models of cell competition loser cells show an increase in P53 expression (Bowling et al., 2018; Lima et al., 2021), combined with the finding that in chimeras *p53* mutant cells contribute preferentially to the embryo (Dejosez et al., 2013), suggests that differences in the levels of P53 expression determine the competitive ability of embryonic cells. To test this possibility, we generated *p53* null mutant mouse ESCs (Fig. 1A) and compared their behaviour when they were cultured in a homogeneous (separate) culture to when they were co-cultured with wild-type cells. We found that in pluripotency culture conditions *p53* mutant cells grew at a similar rate to wild-type cells in separate culture and displayed a small growth advantage in the co-culture condition (Fig. 1B,C). For this experiment, 0.8×10^5^ cells of each genotype were plated in one well of a 12-well plate, in separate cultures. The co-culture contained a mix of 0.4×10^5^ cells of each genotype, totalling 0.8×10^5^ cells per well. In contrast to this, when the same cell numbers were plated and the ESCs were induced to differentiate by culture in neurobasal media (N2B27), *p53^−/−^* cells displayed a small proliferative advantage in separate culture and induced the growth arrest of wild-type cells in co-culture (Fig. 1D,E). These results suggest that mutation of *p53* makes cells into super-competitors that out-compete wild-type cells.

It has been shown that during cell competition pluripotent cells eliminate those cells that initiate differentiation (Díaz-Díaz et al., 2017). The super-competitor behaviour of *p53* mutant cells was not due to the slower differentiation of *p53* mutant cells, as when cultured for 3 days in N2B27 *p53^−/−^* cells downregulate the expression of naïve pluripotency markers and increase the expression of the post-implantation epiblast marker *Fgf5* in a similar way as control cells do (Fig. 1F). Furthermore, at this time point we observed that *p53^−/−^* cells showed similar protein levels of the pluripotency markers OCT4 (POU5F1) and E-cadherin (cadherin 1), as well of the differentiation marker N-cadherin (cadherin 2) (Punovuori et al., 2019), and a trend of increased levels of expression of SOX1 compared with wild-type cells (Fig. S1A-D). Transcriptional profiling by RNA sequencing (RNA-seq) at this time point indicated an enrichment for ‘Signalling pathways regulating pluripotency in stem cells’ (KEGG pathway mmu04550) in the genes downregulated in *p53* mutant cells (Fig. S1E, Table S1; ArrayExpress accession number E-MTAB-13589). This further reinforces the argument that their competitive advantage does not stem from heightened pluripotency.

*p21* (cyclin dependant kinase inhibitor, *Cdkn1a*) is an important target activated by P53 (Pilley et al., 2021). To determine whether P21 regulation of the cell cycle is an important part of the mechanism by which P53 confers a super-competitor status, we generated *p21* mutant ESCs (Fig. S2A). We found that when these cells were cultured in N2B27 they could differentiate normally (Fig. S2B) and grew similarly to control cells in both separate culture and co-culture conditions (Fig. S2C,D), and therefore do not show super-competitor behaviour. The observation that both *p53^−/−^* and *p21*^−/−^ cells displayed similar levels of 5-ethynyl-2′-deoxyuridine (EdU) incorporation to control cells in both separate culture and co-culture with control cells (Fig. 1G, Fig. S2E) further supports the conclusion that regulation of the cell cycle is not the primary mechanism by which *p53* mutant cells become super-competitors.

Given that we did not find evidence for differences in the cycle explaining the increased competitive ability of *p53* mutant cells, we investigated the role of the apoptotic response. When we analysed cleaved caspase 3 expression, we observed that it was significantly lower in *p53^−/−^* ESCs compared with control cells after 3 days and after 4 days culture in N2B27 (Fig. 1H). This indicates that *p53* mutant cells are intrinsically more resistant to apoptosis than are wild-type cells and likely explains their faster growth rate in separate culture. Interestingly, we also observed that wild-type cells at day 4 of co-culture with *p53^−/−^* ESCs showed an increase in cleaved caspase 3 expression compared with when they were maintained in a homotypic (separate) culture (Fig. 1H). The fact that this difference was not apparent at day 3 of co-culture, even though at this time point there is a clear difference in the growth rate of *p53^−/−^* and control ESCs, suggests that there may be caspase 3-independent death occurring before this stage. To address this possibility, we analysed the expression of cleaved caspase 8, which mediates extrinsic cell death signalling (Fuchs and Steller, 2015). We not only found that *p53^−/−^* cells displayed lower levels of cleaved caspase 8 expression than wild-type cells in separate culture (Fig. S3A,B), but also that at day 3 wild-type cells showed higher cleaved caspase 8 expression in co-culture compared with separate culture (Fig. 1I, Fig. S3C). These results suggest that *p53^−/−^* cells out-compete wild-type cells by inducing their apoptotic elimination.

### Developing a mathematical model of super-competition

**Fig. 2.**
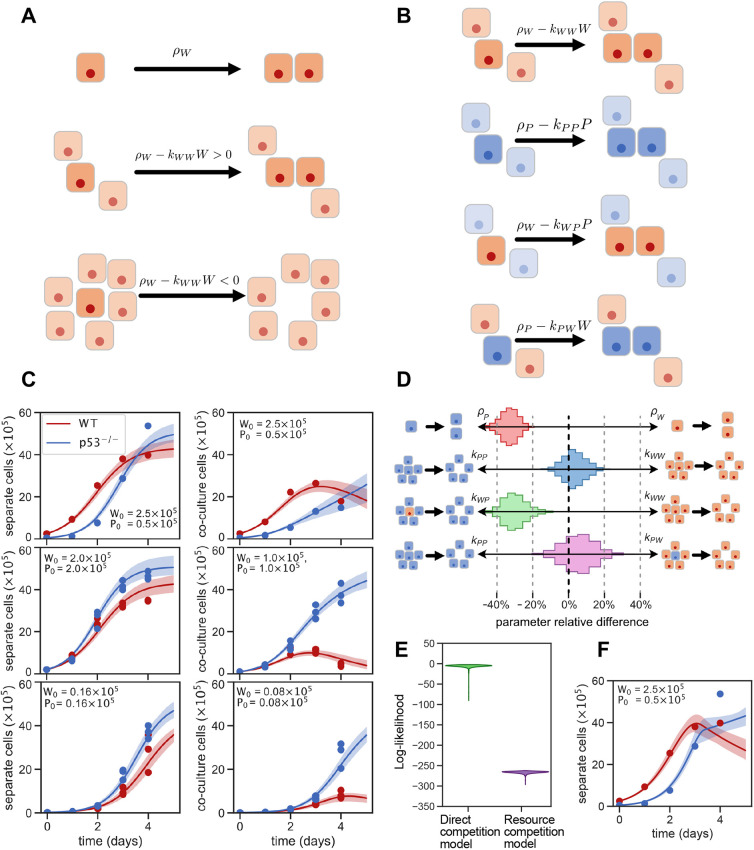
**A mathematical model suggests a direct asymmetric competition.** (A) Schematic depicting how the net proliferation rate per cell in a homotypic population depends on cell density. (B) Schematic showing the four possible direct competition mechanisms affecting population growth of wild-type and *p53^−/−^* cells. (C) Comparison of the experimental data (one circle per replicate) with the model prediction (lines) for the direct competition model (Eqn 1) for nine of 24 of the initial conditions used in the inference (the rest can be found in Fig. S3). Shaded zones show model prediction for the inferred parameter region with likelihood >90% of its maximum. Parameter inference values can be found in Fig. S4. (D) Inferred credibility distributions for the relative differences between different model parameters [distance(p1,p2)=(p2-p1)/(p2+p1)]. Relative differences are significant between the intrinsic growths (*ρ*_*W*_, *ρ*_*P*_) and between the competition constants on wild-type cells (*k*_*WW*_, *k*_*WP*_). (E) Log-likelihood distributions of the direct competition model (Eqn 1) and the resource competition model (Eqn 3). (F) Comparison of the experimental data with the resource competition model; visualisation details are the same as in C. Parameter inference values can be found in Fig. S5. WT, wild type.

Our results indicate that *p53* mutant cells outcompete wild-type cells in co-culture. To gain further insight into this competition and explore the mechanism by which loser cell elimination takes place, we developed a mathematical model to recapitulate quantitatively the differential cell population dynamics in separate and co-culture assays. The aim of this model is twofold: (1) to disentangle potentially confusing effects of population intrinsic growth and cell–cell competition effects on total cell population growth, and (2) to obtain mechanistic information of the nature of competition. We modelled the evolution in time of the number of wild-type cells (*W*) and *p53^−/−^* cells (*P*) by specifying a set of ordinary differential equations (ODEs) describing the effect that different population compositions (*W*, *P*) have on the net growth of each species. In the absence of competition – at low cell numbers – each species population grows with an intrinsic rate *ρ_i_*>0, *i*={*W,P*} (Fig. 2A, top); this is the net growth of the population incorporating the intrinsic proliferation and apoptotic rates. As the population grows, cells compete with each other with a strength *k_j_* specific for each of the four possible interactions (*j*={*WW,WP,PW,PP*}; see Fig. 2A,B):
(1)

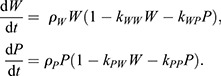
This Lotka–Volterra description has been successfully used in a different context of cell competition (Ram et al., 2019) and assumes that the magnitude of the competition is proportional to the number of cells of each type in the dish (or, equivalently, their concentration). In particular, the *k_j_* parameters control apoptosis owing to crowded cell populations and accommodate for the possibility of different apoptotic rates for each cell type in homotypic (*k_WW_* and *k_PP_*) and heterotypic (*k_WP_* and *k_PW_*) environments (Fig. 2A,B). This increase in apoptotic rate due to cell competition is translated into a decrease of the total net growth rate of the population of each species, making it negative in crowded environments (e.g. 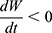; see Fig. 2A). To test the model, we used Bayesian inference to identify which portion of the parameter space {*ρ_i_*,*k_j_*} is consistent with the experimental data by using a likelihood function that compares experimental and numerical trajectories (see Materials and Methods). The initial parameter space explored (the prior distribution) included parameter regions equally compatible with different hypotheses. This distribution will be constrained by the experimental data resulting in parameter distributions (the posterior distribution) that can be used to perform hypothesis testing (e.g. we can study whether intrinsic proliferation rates differ from each other by analysing the fraction of the posterior distribution in which *ρ*_*P*_>*ρ*_*W*_). The result of this analysis is a preliminary distribution of the parameters of the model compatible with the cell population trajectories shown in Fig. 1. Inspection of this distribution of parameters was not enough to address different hypotheses, but allowed us to identify which additional experimental initial plating conditions would carry information to improve our predicted posterior parameter distributions. Experiments were then performed using these identified plating conditions and were analysed in the same manner as described above. This resulted in an iterative analysis composed of a set of experimental trajectories for 24 additional different plating conditions that generated a posterior credibility distribution of the parameters that can be used to extract mechanistic information of the competition (Fig. S4). The trajectories corresponding to this final distribution show a very good fit to the model (Fig. 2C). Most importantly, the resulting distributions allow us to compare different hypotheses through the pairwise relationships between parameters. This can be done by evaluating the extent of parameter regions that satisfy inequalities between different parameters (Fig. 2D, Fig. S5). This confirmed that the intrinsic net growth of *p53^−/−^* is faster than the intrinsic net growth of wild-type cells, *ρ*_*P*_>*ρ*_*W*_ (Fig. 2D). In addition, comparison of the competition strengths revealed that there were no significant differences between the competition of *p53^−/−^* cells with themselves compared with the corresponding homotypic competition between wild-type cells (*k*_*WW*_≃*k*_*PP*_). However, as expected, the presence of *p53^−/−^* ESCs induced a dramatic decrease on the growth rate of wild-type cells (*k*_*WW*_<*k*_*WP*_). Strikingly, *p53^−/−^* ESC growth was unaffected by the identity of the cells in their neighbourhood (*k*_*PP*_≃*k*_*PW*_). Our model therefore quantifies the interactions between cells allowing us to identify differential cellular properties and compare their magnitude.

### *P53* mutant cells induce the apoptotic elimination of wild-type ESCs

Our model indicates that *p53^−/−^* ESCs have a direct negative effect on wild-type cells, and the increased expression of apoptotic markers in co-cultured wild-type cells (Fig. 1H,I, Fig. S3A-C) suggests that this takes place through the induction of their apoptotic elimination. To test this possibility, we first performed the cell competition assays in the presence of a pan-caspase inhibitor (Z-VAD-FMK). For this, we cultured wild-type and *p53^−/−^* ESCs in N2B27 in separate culture and co-culture conditions and added the caspase inhibitor from day 2 to day 4 of culture. We found that this partially rescued the elimination of wild-type cells in co-culture (Fig. S3D), raising the prospect that non-apoptotic forms of cell death may be contributing to the out-competition of wild-type cells.

**Fig. 3.**
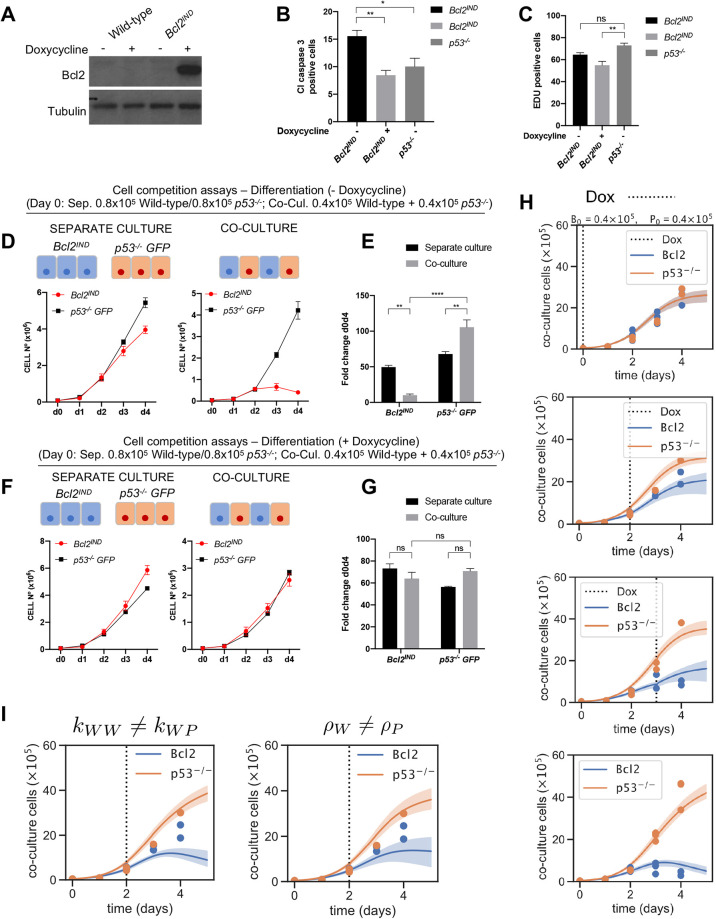
***p53^−/−^* ESCs induce the apoptotic elimination of wild-type cells in co-culture.** (A) Bcl2 levels in wild-type and *Bcl2*^*Ind*^ cells not treated or treated with doxycycline for 3 days. (B) Percentage of cleaved caspase 3-positive cells in *Bcl2*^*Ind*^ treated with or without doxycycline and *p53^−/−^* cells. (C) Percentage of EdU incorporation in *Bcl2*^*Ind*^ treated with or without doxycycline and *p53^−/−^* cells. (D) Growth curves of *Bcl2*^*Ind*^ and *p53^−/−^* cells over 4 days in separate or co-culture conditions (as indicated by schematics) without doxycycline treatment. (E) Fold change in wild-type and *p53^−/−^* cell numbers between day 0 and day 4. (F) Growth curves of *Bcl2*^*Ind*^ and *p53^−/−^* cells over 4 days in separate or co-culture conditions with doxycycline treatment from day 1. (G) Fold change in wild-type and *p53^−/−^* cell numbers between day 0 and day 4. (H) Comparison of the direct competition model (lines) with the experimental data (one replicate per circle) for doxycycline treatment at different days (vertical dashed lines). Shaded zones show model prediction for the inferred parameter region with likelihood >90% of its maximum. Parameters are the same as in Fig. 2, Figs S3, S4. Data were obtained from three independent experiments and are shown as the mean+s.e.m. (E-H). (I) Resulting model trajectories when doxycycline-treated cells are simulated by only changing the intrinsic growth and maintaining differences in competition strength (left), or only changing the competition strength and maintaining differences in intrinsic growth (right). *P<0.05, **P<0.01. d, days; ns, not significant.

BCL2 is a key anti-apoptotic protein acting in the mitochondrial apoptotic pathway (Tait and Green, 2013) and *Bcl2* overexpression prevents loser cell elimination in interspecies chimeras (Zheng et al., 2021). We therefore generated doxycycline-inducible ESCs (Fig. 3A). Induction of BCL2 expression by doxycycline addition decreased the intrinsic apoptotic rate to levels that were similar to those found in *p53* mutant cells (Fig. 3B). In contrast, BCL2 induction did not significantly affect proliferation rates (Fig. 3C). We therefore assayed the behaviour of *Bcl2*-inducible ESCs (*Bcl2^Ind^*) in separate culture and co-culture with *p53^−/−^* cells. We found that, similarly to what occurred with wild-type ESCs, when *Bcl2^Ind^* ESCs were cultured separately in N2B27 without the addition of doxycycline they grew slightly slower than *p53^−/−^* ESCs, and when co-cultured with *p53^−/−^* ESCs they were effectively eliminated (Fig. 3D,E). In contrast, we observed that upon BCL2 induction with doxycycline *Bcl2^Ind^* ESCs grew similarly to *p53^−/−^* cells, both in separate culture and co-culture conditions (Fig. 3F,G) and were therefore no longer eliminated by *p53^−/−^* cells. Importantly, the longer the cells were treated with doxycycline, the better the rescue was: co-cultures treated from day 0 showed a complete rescue, adding doxycycline from day 2 reduced the level of rescue, and adding it from day 3 reduced the rescue further (Fig. 3H). These results indicate that *p53* mutant cells out-compete wild-type cells by inducing their apoptotic elimination.

To analyse the dynamics by which BCL2 rescues the competition phenotype, we used our model together with the inferred parameters to reproduce experimental trajectories of doxycycline-induced rescue at different time points of the competition. The model successfully reproduced the dynamics of the rescue (Fig. 3H). Untreated *Bcl2^Ind^* ESCs were successfully simulated using the same parameters as wild-type cells. In contrast, reproduction of the experimental trajectories of *Bcl2^Ind^* cells treated with doxycycline required complete elimination of the asymmetry in competition, reducing the competition strength that *p53* mutant cells have on *Bcl2^Ind^* (*k*_*WP*_) to the same level as homotypic competition (*k*_*WW*_=*k*_*WP*_) (Fig. 3I). Interestingly, it also required elimination of the asymmetry in the intrinsic population growth by increasing the net proliferation rate of *Bcl2^Ind^* cells to the same level as *p53^−/−^* ESCs (*ρ*_*W*_=*ρ*_*P*_). In summary, BCL2 rescue dynamics could only be reproduced by the model when all the parameters of wild-type cells were restored to the equivalent parameters of *p53^−/−^* ESCs, supporting the hypothesis that the competition mechanism is an active elimination of wild-type cells by *p53^−/−^* cells.

### Short-range signalling mediates the elimination of wild-type cells by *p53*^−/−^ ESCs

**Fig. 4.**
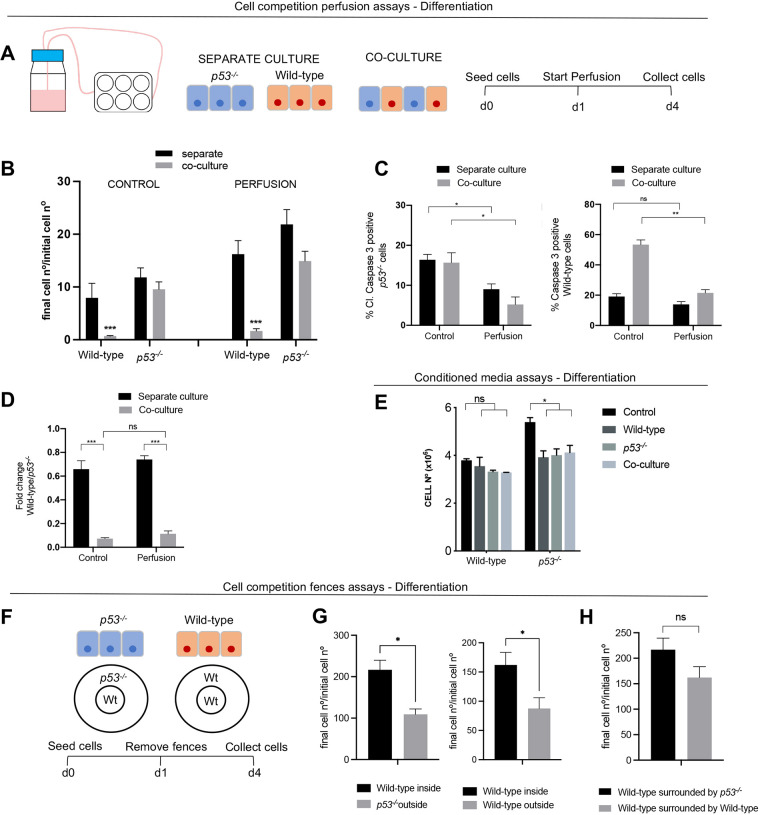
**Wild-type cell elimination is cell contact dependent.** (A) Schematic experimental setup whereby cells were cultured separately and together and N2B27 media was perfused over the plate from day 2 until day 4. Cells were then counted and fixed. (B) Wild-type and *p53^−/−^* final/initial cell numbers over 4 days cultured separately or together in a control plate and in a perfused plate. (C) Percentage of cleaved caspase 3 in wild-type and *p53^−/−^* cells cultured separately or in co-culture with and without perfusing N2B27 media. (D) Fold change in wild-type cell numbers between day 0 and day 4 when cultured alone or with *p53^−/−^* cells in N2B27 in a control plate and in a perfused plate. (E) Cell numbers of wild-type and *p53^−/−^* cells cultured separately for 4 days and adding conditioned media from day 3 to day 4. Conditioned media was taken from cultured wild-type and *p53^−/−^* cells or from a co-culture of both cell types. (F) Schematic experimental setup whereby wild-type cells where cultured surrounded by *p53^−/−^* cells or wild-type (Wt) cells in the same well without cell contact between both cell types. (G) Fold change in wild-type and *p53^−/−^* cell numbers between day 0 and day 4. (H) Fold change in wild-type cell numbers a between day 0 and day 4 when they are cultured surrounded by wild-type or *p53^−/−^* cells. Data were obtained from three independent experiments and are shown as the mean+s.e.m. *P<0.05, **P<0.01. d, days; ns, not significant.

The data presented in Figs 1–3 point to a direct competition between *p53^−/−^* and wild-type cells, whereby one induces the elimination of the other. But, in addition to the direct competition model (Eqn 1), we also wanted to explore the possibility that cells compete for a shared resource that is consumed over time with differential rates and tolerances depending on the cell type (see Materials and Methods; Eqn 3). Interestingly, this model was not able to recapitulate the experimental growth curves (Fig. 2E,F, Fig. S5). To test this assumption experimentally, we analysed the effect that providing unlimited amounts of nutrients has on the dynamics of the competition between wild-type and *p53^−/−^* cells. For this, we performed our competition experiments in a culture media that was continuously perfused from day 1 of culture by a pump (Fig. 4A). Using this system, we observed that both wild-type and *p53^−/−^* ESCs grew at roughly twice the rate when the media was perfused compared with when it was not, in both separate culture and co-culture conditions (Fig. 4B). This increase in growth was likely due to a lower level of cell death, as the percentage of cells that were positive for cleaved caspase 3 was also significantly reduced in the perfusion condition (Fig. 4C). It is also possible that the apoptotic cells are being more efficiently removed by the perfusion. Notably, we found that, despite these lower levels of apoptosis, the perfusion did not significantly affect the degree to which wild-type ESCs were eliminated in co-culture (Fig. 4B,D), suggesting that the latter may be true. These results suggest two things. First, they indicate that the elimination of wild-type cells by *p53^−/−^* ESCs is unlikely to be due to nutrient deprivation. Second, the fact that the number of cells in the culture dish can double without increasing the degree of wild-type apoptosis, indicates that wild-type cells can become more packed without this increasing their rate of elimination. This suggests that their elimination is not due to mechanical stress, as has been shown to be the case for MDCK cells with increased p53 expression (Wagstaff et al., 2016). In support of this possibility, we find two further things. First, when 0.4×10^5^, 0.8×10^5^, 2×10^5^ and 10×10^5^ cells are seeded, and the rate of wild-type elimination is calculated between days 3 and 4 of differentiation, this rate of elimination is highest at the 2×10^5^ seeding density, but then decreases to 60% those rates for 10×10^5^ cells seeded (Table S2). Similarly, when 0.4×10^5^, 0.8×10^5^, 1.6×10^5^ and 3.2×10^5^ cells are seeded, normalised caspase 8 levels in wild-type cells peak at the 1.6×10^5^ seeding density and then decrease when 3.2×10^5^ cells are seeded (Fig. S6). The lack of a direct correlation between cell density and cell death in wild-type cells strengthens the argument that the elimination of wild-type cells is not due to mechanical stress.

We next analysed the possibility that diffusible growth factors secreted into the media could be inducing the elimination of loser cells. For this, we did two things. First, we analysed the effect of conditioned media taken from wild-type and *p53* mutant cells cultured separately, as well as well as taken from their co-culture. We observed that none of these conditioned medias had any effect on the growth of wild-type cells and they all reduced *p53^−/−^* cell growth (Fig. 4E). To address further the importance of secreted factors, we used a fences system (Lawlor et al., 2020), whereby one population of cells is grown surrounded by another but separated by fences that are removed once the cells are seeded (Fig. 4F). This allows the different cell populations to be cultured without contact but sharing the same media. When this was done, we observed that cells grown on the outside layer grew slower than those cultured on the inside, irrespective of their genotype (Fig. 4G). Importantly, we found that wild-type cells grew similarly if they were surrounded without contact by *p53* mutant cells or by other wild-type cells (Fig. 4H). Together, these data suggest that cell–cell contact or short-range signalling is required for *p53^−/−^* cells to eliminate wild-type cells.

**Fig. 5.**
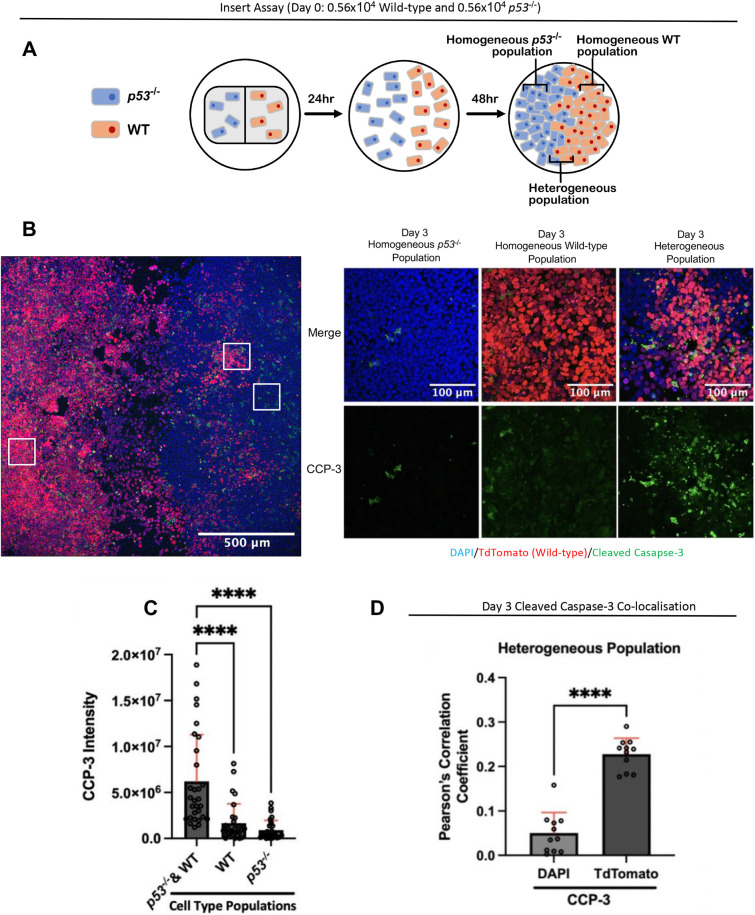
**Short-range signalling mediates loser cell elimination.** (A) Schematic of the fence assay experimental setup within one well, illustrating the heterogeneous and homogeneous regions from which fluorescence measurements were taken. (B) Images of cleaved caspase 3 (CCP-3) immunostained wild-type and *p53^−/−^* ESCs in homogeneous and mixed regions at day 3 of differentiation in N2B27. Wild-type ESCs are labelled with TdTomato (red) and DAPI; *p53^−/−^* ESCs are only DAPI positive. (C) Quantification of CCP-3 intensity from heterogeneous *p53^−/−^*/wild-type ESC populations, as well as from homogeneous populations. Bars indicate mean±s.d. A Kruskal–Wallis test was performed followed by Dunn's multiple comparisons. ****P<0.0001, *n*=4, *N*=30 for all conditions. (D) Colocalisation of CCP3- and TdTomato-labelled wild-type ESC nuclei and DAPI-labelled wild-type/p53^−/−^ nuclei, expressed as Pearson's coefficient. Colocalisation was performed in heterogeneous *p53^−/−^*/wild-type regions. Statistical comparison performed using an unpaired two-tailed *t*-test, ****P<0.0001, *n*=11 for all conditions. WT, wild type.

To test the possibility discussed above, we seeded wild-type cells and *p53^−/−^* cells into two culture wells separated by an insert (Fig. S7A). The insert was removed, and the cells were allowed to come together. When this was done, we observed areas where the cells were mixed and others that were either wild-type or *p53^−/−^* (Fig. 5A,B). Analysis of cleaved caspase 3 expression revealed that cell death levels were significantly higher in the mixed area than in regions that were predominantly populated by wild-type or *p53^−/−^* cells (Fig. 5B,C). Furthermore, the apoptosis observed in the mixed region primarily occurred in wild-type cells (Fig. 5C). This supports the requirement for contact or short-range signalling for the elimination of wild-type loser cells.

### Cell neighbourhood changes during cell competition

**Fig. 6.**
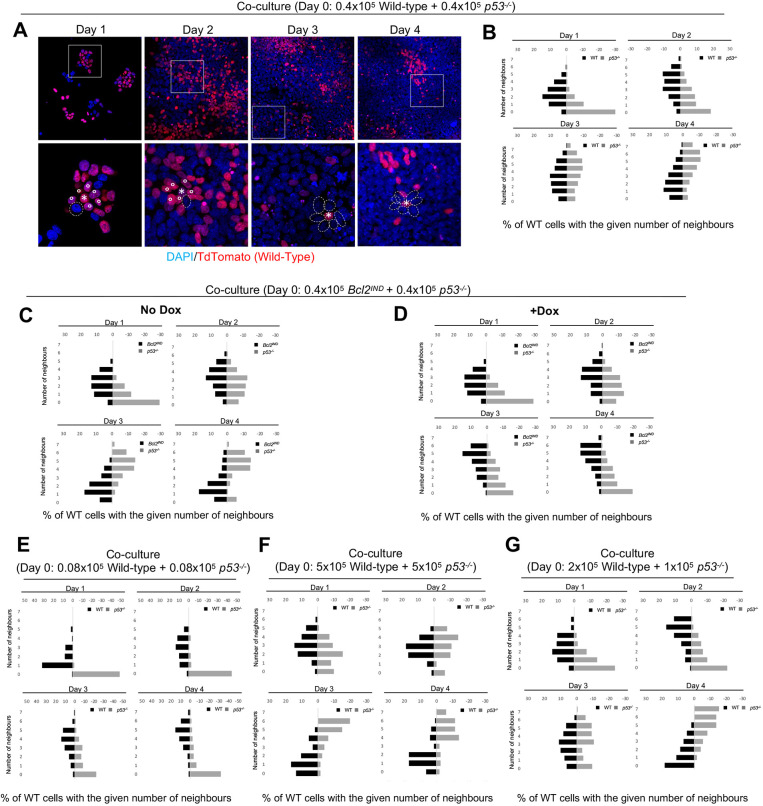
**Neighbourhood changes during cell competition.** (A) Immunostaining of wild-type and *p53^−/−^* cells co-cultured from day 1 to day 4 of the culture. An example of neighbours of wild-type cells is shown for each day. Boxed regions are shown at higher magnifications below. Asterisks indicate wild-type cells, continuous lined circles indicate wild-type neighbours and dashed line ovals indicate p53^−/−^ neighbours. (B) Number of wild-type and *p53^−/−^* neighbours of wild-type cells from day 1 to day 4 of the co-cultures. (C) Number of *Bcl2^IND^* and *p53^−/−^* neighbours of *Bcl2^IND^* cells from day 1 to day 4 of co-cultures without doxycycline treatment. (D) Number of *Bcl2*^*Ind*^ and *p53^−/−^* neighbours of *Bcl2*^*Ind*^ cells from day 1 to day 4 of co-cultures with doxycycline treatment from day 1. (E) Number of wild-type and *p53^−/−^* neighbours of wild-type cells from day 1 to day 4 of co-cultures with lower initial cell numbers plated. (F) Number of wild-type and *p53^−/−^* neighbours of wild-type cells from day 1 to day 4 of co-cultures with higher initial cell numbers plated. (G) Number of wild-type and *p53^−/−^* neighbours of wild-type cells from day 1 to day 4 of co-cultures with different percentages of seeded cells. Data were obtained from three independent experiments and are shown as the mirrored distribution of wild-type and *p53^−/−^* neighbour numbers. WT, wild type.

Given the importance of short-range signalling for the elimination of wild-type cells, we investigated whether changes in the cell neighbourhood could explain the competition between wild-type and *p53* mutant cells. We consider here the local cell neighbourhood to be the relative numbers of wild-type and *p53^−/−^* cells that are in direct contact with a given cell in the co-culture condition. When we analysed the cell neighbourhood of wild-type cells, we found that during the timecourse of the experiment these cells increased the average number of wild-type neighbours until reaching a peak at day 2 of co-culture, but that after this time point the number of wild-type neighbours decreased (Fig. 6A,B). In contrast, the average number of *p53^−/−^* neighbours that wild-type cells had increased from day 1, and by day 3 they had more *p53^−/−^* neighbours than wild-type neighbours. This switch in wild-type neighbourhood between days 2 and 3 coincided with when wild-type elimination is most obvious in the growth curves of these cells (Fig. 1C). The same neighbourhood dynamics could be observed for *Bcl2^Ind^* cells when these were co-cultured with *p53^−/−^* ESCs without doxycycline (Fig. 6C). However, when BCL2 was induced with doxycycline and loser cell elimination prevented, then *Bcl2^Ind^* cells showed a daily increase in *Bcl2^Ind^* cell neighbours, whereas the number of *p53^−/−^* ESC neighbours first increased until day 2 and then decreased (Fig. 6D). These results are consistent with cell neighbourhood having a role in the outcome of competition.

We next tested the effects of changing the concentrations of cells plated. First, we analysed the effects of reducing the cell numbers plated. For this, a mixture of 0.08×10^5^ wild-type cells and 0.08×10^5^ *p53^−/−^* ESCs were plated in the co-culture condition. We observed that at this low confluency there was no substantial change in the neighbourhood of wild-type cells in co-culture (Fig. 6E). Interestingly, this correlated with the total number of wild-type cells still increasing at day 4 of co-culture (Fig. S7A) and the lowest rate of their elimination between days 3 and 4 of all conditions tested (Table S2). This suggests that the low confluency is reducing the number of possible *p53^−/−^* neighbours, and that this is decreasing the rate of competition. We then analysed the effects of increasing the cells numbers plated at day 0 to a mixture of 5×10^5^ wild-type and 5×10^5^ *p53^−/−^* cells in co-culture. This condition led to wild-type cells having more *p53^−/−^* cell neighbours from day 2 (Fig. 6F) and being robustly eliminated in co-culture (Fig. S5B). Similar effects were observed when we changed the proportion of wild-type to *p53^−/−^* cells plated. When a mixture of 2×10^5^ wild-type and 1×10^5^ *p53^−/−^* cells were plated in co-culture this caused a shift between days 2 and 3 in the neighbourhood of wild-type cells from having more wild-type neighbours to having more *p53^−/−^* ones (Fig. 6G). This correlated with the timings of wild-type cell elimination in co-culture (Fig. S5C). These results, together with the importance of short-range signalling identified in Fig. 4 and Fig. S7, point to the direct interaction between wild-type and *p53^−/−^* cells determining the outcome of their competition.

To test further the importance of the relative number of neighbours for wild-type cell elimination, we plated cell competition experiments with the following ratios of wild-type and *p53^−/−^* cells: 1:1; 1:3; 1:5 and 1:10. We then calculated the rate of elimination per day and between conditions. We observed that the rate of loser cell elimination increased with time and was highest between days 3 and 4 (Table S3). Furthermore, we also observed that as the proportion of wild-type cells was diluted the rate of elimination increased (Table S3), suggesting that a higher proportion of winner neighbours accelerates the elimination of losers. These data support the hypothesis that the relative neighbourhood of loser cells is a factor regulating their elimination.

## DISCUSSION

Cell competition is a fitness quality-control mechanism that eliminates cells that are less fit than their neighbours. One important implication of cell competition is that the fitness of a cell is relative to the fitness of its neighbour. For example, in mouse, wild-type cells eliminate cells with mitochondrial dysfunction (Lima et al., 2021), but are eliminated by cells that overexpress MYC (Clavería et al., 2013; Sancho et al., 2013). Here, we have addressed how the expression levels of P53 affects the competitive nature of pluripotent cells. We find that cells lacking *p53* behave as super-competitors and eliminate their wild-type neighbours. This elimination is dependent on the onset of differentiation, as when cells are cultured in pluripotency conditions mutation of *p53* does not provide cells with a competitive advantage. Furthermore, by combining mathematical modelling and cell-based assays we also show that the competitive advantage of *p53* null mutant cells is mediated by short-range signalling interactions that induce the elimination of wild-type cells, rather than through competition for nutrients or space. This highlights the importance of the local cell neighbourhood for the regulation of proliferation during early embryonic development.

Over the last few years, there has been increasing evidence for a key role for P53 in cell competition. In *Drosophila*, p53 has been shown to be required in Myc-overexpressing super-competitor cells to induce wild-type cell elimination (de la Cova et al., 2014). In these winner cells, p53 regulates metabolism by promoting oxidative phosphorylation and inhibiting glycolysis. In contrast, mechanical stress induces increased *p53* expression in polarity-deficient MDCK cells and this activation causes the elimination of these loser cells (Wagstaff et al., 2016). In mouse cell competition, p53 has been shown to play multiple roles. In haemopoietic stem/progenitor cells, DNA damage causes the out-competition of cells with higher p53 levels (Bondar and Medzhitov, 2010). Here, the damage induces senescence in those cells that have higher p53 levels than their neighbours. In the mouse embryo, it was found that clones carrying a mutation of *Mdm2/4*, and therefore have elevated P53 expression, are outcompeted by wild-type cells (Zhang et al., 2017). Similarly, we have shown that during the onset of differentiation, defective cells, such as cells with impaired BMP signalling or tetraploid ESCs, show increased p53 expression that is required for their out-competition by wild-type cells. We also demonstrated that the mechanism of elimination of these defective cells is because in a competitive environment p53 represses mTOR signalling and this induces apoptosis (Bowling et al., 2018). Importantly, BMP signalling-defective and tetraploid cells are eliminated because of their mitochondrial dysfunction and mutation of *p53* prevents this elimination and restores mitochondrial membrane potential in these cells (Lima et al., 2021). Also in mouse ESCs, prior to differentiation, p53 has been shown to regulate mitochondrial membrane potential and oxidative phosphorylation by controlling PUMA (BBC3) and NOXA (PMAIP1) expression, and mutation of *p53* also provides cells with a competitive advantage in this context (Valverde-Lopez et al., 2023 preprint). Our results presented here, together with these studies, suggest therefore that p53 could act as a general sensor of cell fitness across different tissues.

Three main modes of cell competition have been proposed: competition for nutrients, mechanical competition, and direct fitness sensing between cells (Bowling et al., 2019). For example, during cell competition in MDCK cells, elevated p53 expression sensitises cells to compaction, indicating that differences in p53 expression determine a differential response to mechanical stress (Wagstaff et al., 2016). In *Drosophila*, p53 regulates cell metabolism to determine the competitive nature of cells in the imaginal wing disc (de la Cova et al., 2014). This metabolic role could be used to infer that p53 is required to establish differences in nutrient metabolism that will in turn direct the outcome of competition. However, our studies described here analysing the behaviour of *p53* mutant pluripotent cells suggest that during ESC differentiation p53 is possibly playing roles that are different to those described above for MDCK cells and in *Drosophila*. Our observation that there is no change to the cell competition dynamics between wild-type and *p53^−/−^* ESCs despite cell numbers doubling when assays are performed in media that is being continuously replenished through cell perfusion suggests that neither nutrient nor space availability is determining the outcome of the competition between these cell types. The observation that a fivefold increase in the number of cells seeded, from 0.2×10^6^ to 1×10^6^, decreases the rate of wild-type cell elimination rather than increasing it supports this argument. Instead, our findings reveal that a change in the cell neighbourhood of loser wild-type cells is correlated with the outcome of cell competition. This suggests that the local interaction between wild-type and mutant cells regulates the elimination of the wild-type cells. These results are in line with what has been observed with *Myc*-overexpressing ESCs, which eliminate wild-type cells via short-range/contact-dependent signalling (Díaz-Díaz et al., 2017) and contrast with our own findings that the elimination of ESCs with defective BMP signalling is mediated by long-range signalling (Sancho et al., 2013). Our observations that the relative number of winner/loser neighbours determines the outcome of cells competition are also in accordance with what has been found in the *Drosophila* pupal notum, where the super-competition ability of *Myc*-overexpressing cells has been related to the relative surface area shared between winner and loser cells (Levayer et al., 2015). This suggests that replacement of wild-type cells in a tissue occurs through a different mechanism than the replacement of dysfunctional cells, and therefore during embryonic development there may be several distinct forms of cell competition acting in a tissue at the same time.

Computational and mathematical modelling has provided invaluable insight allowing us to distinguish between possible models of competition, as well as revealing and quantifying the minimal rules required to reproduce the competition dynamics observed. Further descriptions of local competition in confluent populations – where the population cannot be considered homogeneous – will require a mathematical framework that incorporates the spatial dynamics of the competition. Such models can use similar ODE formulations where clone shape is taken into account implicitly in the functional form of the competition terms (Nishikawa et al., 2016). Alternatively, more detailed descriptions of the cellular monolayer can be simulated by using a computational vertex model, where the dynamics and environment of each individual cell are taken into account. These spatial models would allow us to study additional modes of competition resulting from dynamics exclusive to confluent tissues. For example, interaction between cell populations growing at different rates has been shown to introduce mechanical stress that can act as a feedback mechanism to stabilise uniform growth (Shraiman, 2005). Similarly, cell geometry and mechanical heterogeneities within a developing tissue can bias mechanical cell elimination, leading to effective changes in competition properties of co-existing cellular populations (Lee and Morishita, 2017). In addition, following cell elimination, cell type-specific topological remodelling of epithelial junctions has been shown to be enough to induce a difference in the fitness between cell populations in the *Drosophila* wing disc (Tsuboi et al., 2018).

More sophisticated agent-based multi-scale models can be used to simulate more specific cell and tissue morphologies, such as competition during the transition to congruence. An example of such models was used by Gradeci et al. (2021) using automatic annotation of movies of co-cultured wild-type and polarity-deficient MDCK cells lasting up to 4 days, providing extensive input on the behaviour of wild-type and mutant cells. Their modelling identified that cell density and stiffness is sufficient to account for the apoptotic elimination of loser cells during mechanical competition. In contrast, the outcome of biochemical competition appears to be primarily regulated by the organisation of winner and loser cells in the tissue. Interestingly, these results are very much in accordance with the likely role of cell neighbourhood identified in our study. One of the main limitations of the successful application of spatial computational models is the large number of parameters and rules that can be used to describe the behaviour of cellular populations, making hard to infer confidently details of the model even in simple scenarios (Kursawe et al., 2018). This highlights the necessity of minimal models able to incorporate and test mechanistic hypotheses matching the complexity of the available data.

In conclusion, our studies identify that upon exit from pluripotency loss of p53 expression is sufficient to make ESCs into super-competitors. These winner cells induce the replacement of healthy wild-type cells via a direct induction of apoptosis. This cell replacement not only provides a potential explanation for the expansion of cells with *p53* mutations in human pluripotent stem cell cultures (Merkle et al., 2017), but, importantly, together with our finding that those cells eliminated in the early mouse embryo have a signature of elevated p53 expression (Lima et al., 2021), suggest that competitive interactions between cells with different levels of p53 expression shape growth during development.

## MATERIALS AND METHODS

### Cell lines used and cell line generation

To generate *p53^−/−^* cells, a p53 sgRNA (5′-GCAGACTTTTCGCCACAGCG-3′) was cloned into a lentiCRISPRv2 vector. This targets position 775/exon 6 in the transcript and amino acid 203 in the protein sequence, interrupting the p53 DNA-binding domain. Viruses were generated by transfecting this vector along with helper plasmids VSV-G and psPAX2 into HEK293T packaging cells. After 48 h, the media from these cells was applied to mouse ESCs with 4 µg/ml polybrene. Two rounds of infection, one for 4 h and then one overnight, were carried out. The cells were then selected using 2 µg/ml puromycin and plated at single-cell confluency. Clones were screened for loss of p53 protein by western blot. Two different clones were used in the experiments described in Fig. 1 and the rest of experiments were performed with clone 1.

To generate *p21^−/−^* cells, a p21 sgRNA (5′-GATTGCGATGCGCTCATGGC-3′) was cloned into the px330 vector (Addgene, 158973). ESCs were co-transfected with 2 µg of this vector and 0.12 µg of a hygromycin marker (631625, Takara Bio) using Lipofectamine 2000 (Invitrogen) according to manufacturer's instructions. The cells were selected using 150 µg/ml hygromycin and plated at single-cell confluency. Clones were screened for loss of p21 protein by western blot.

To generate *Bcl2^Ind^* cells, mouse ESCs were co-transfected with 1 µg pPB-TRE(3G)-hBCL2-PURO, 1 µg pPB-CAG-rtTA(3G)-NEO and 1 µg pCMV-PBase using Lipofectamine 2000 (Invitrogen) according to manufacturer's instructions. The cells were selected using 1 µg/ml doxycycline, 2 µg/ml puromycin and 300 µg/ml neomycin and plated at single-cell confluency. Clones were screened by western blot for BCL2 protein overexpression upon 1 µg/ml doxycycline treatment.

The H2B-tdTomato ESCs were a kind gift of Professor Jenny Nichols (University of Edinburgh, UK) and were considered wild type for the purpose of the cell competition experiments described.

The identity of all cell lines and their contamination status was checked at regular intervals.

### Cell culture

All cells were cultured at 37°C in an atmosphere with 5% CO_2_. Reagents used for tissue culture were obtained from Invitrogen unless otherwise stated. Mouse ESCs were cultured on 0.1% gelatin-coated flasks (Nunc, Thermo Fisher Scientific) in GMEM containing 10% (v/v) foetal calf serum (FCS; Seralab), 1× non-essential amino acids, 2 mM L-glutamine, 0.1 mM β-mercaptoethanol and supplemented with homemade leukaemia inhibitory factor (LIF; 1:1000). ESCs were routinely dissociated with trypsin and cryopreserved in 10% DMSO in FCS.

### Competition assay

Cells were seeded onto glass coverslips placed on plates that were then coated with fibronectin (Merck) at a concentration of 2.5×10^4^ cells/cm^2^ either separately or mixed for co-cultures at a 50:50 ratio (Sancho et al., 2013). Cells were cultured in N2B27 media (DMEM F12 media, 0.5× B27 supplement; 0.5× N2 supplement; 0.1 mM 2-mercaptoetanol, 2 mM glutamine; all Thermo Fisher Scientific) for 3-4 days to allow for differentiation. At the indicated time points, the cells were counted using a Vi Cell Counter and Viability Analyser (Beckman Coulter) and proportions of each cell type in co-cultures were determined using LSR II Flow Cytometer (BD Bioscience). Sytox Blue (Thermo Fisher Scientific) or propidium iodide (Sigma-Aldrich) was used to stain for dead cells.

### Growth rates and cell elimination rates

Growth rates were calculated by dividing the cell number of one day by the cell number of the previous day Table S2. The elimination rate was calculated by dividing the cell number of one day by the cell number of the next day (Table S2) or by dividing the proportion of wild-type cells present one day by the proportion present the next (Table S3).

### Perfusion assay

Cells were seeded into 6-well perfusion plates (AVP011, Reprocell) coated with fibronectin either separately or mixed for co-cultures at a 50:50 ratio in N2B27 media. At day 1, the plate was connected to a bottle with 200 ml N2B27 and perfused using a Watson Marlow 120 U pump with a speed of 9 rpm. The media circulated from the bottle to the plate, where each well is connected to the next one by a channel, so a unidirectional flow of media was established to each well, and then back to the bottle. The culture was perfused until day 4, when the cells were counted or fixed.

### Fences and conditioned media assays

For the fence assays, fences (Aix-Scientifics) were placed in each well of a 24-well plate coated with fibronectin. 0.008×10^6^ cells were seeded in the inner ring and 0.035×10^6^ cells in the outer ring. The fences were removed the following day and the media was replaced every day. At the indicated time points, the fences were replaced in the well to count cell numbers using a Vi Cell Counter and Viability Analyzer (Beckman Coulter).

For the culture well insert assay, two well culture inserts (Ibidi) were placed into each well of an 8-well chamber slide (LabTek) coated with fibronectin (1:100, Merck) and 2.55×10^4^ cells/cm^2^ of the respective cell type were seeded into each well of the culture insert and cultured with N2B27 differentiation media. Culture inserts (Ibidi) were removed after 24 h and N2B27 media was replenished every 24 h, until immunofluorescence staining was performed.

For conditioned media assays, 0.08×10^6^ wild-type or *p53^−/−^* cells were seeded and from day 2 cells were cultured with conditioned media obtained from wild-type or *p53^−/−^* cells cultured separately or from a co-culture. Conditioned media was obtained from the corresponding cell types and was concentrated using Vivaspin 500 centrifugal concentrators (GE Healthcare) according to the manufacturer's instructions.

### Flow cytometry staining

Cells were detached from the plates using Accutase (Sigma-Aldrich) and fixed in 7.4% formaldehyde in N2B27 media for 10 min. Permeabilisation was carried out using ice-cold methanol and cells were blocked using 1% bovine serum albumin (BSA). Cells were then incubated with primary antibody (cleaved caspase 3 9664, Cell Signaling Technology; 1:200) for 1 h at room temperature. After washing, cells were incubated with the secondary antibody (Alexa Fluor 546/405, Thermo Fisher Scientific; 1:2000) for 30 min at room temperature. Flow cytometry was performed using an LSR II Flow Cytometer and analysed using FlowJo software v9 or v10.0.7r2 (BD Bioscience).

### Proliferation assay

Cells were incubated with EdU from the Click-iT kit (Thermo Fisher Scientific) for 2 h according to the manufacturer's instructions. Cells were then detached from plates using Accutase and analysed by flow cytometry using an LSR II Flow Cytometer and FlowJo software.

### Stem cells immunofluorescence

For immunostaining, mouse ESCs and epiblast-derived stem cells were fixed for 10 min in 4% paraformaldehyde at room temperature, permeabilised in 0.4% Triton X-100 in PBS for 5 min at room temperature, blocked in 10% BSA, 0.1% Triton X-100 in PBS and incubated overnight at 4°C in primary antibody diluted in 1% BSA, 0.1% Triton X-100 in PBS [anti-cleaved caspase 3 (Asp175, Cell Signaling Technology; 1/100); anti-Nanog (14-5761-80, eBioscience; 1/100), anti ATP-b (Ab14730, Abcam; 1:200) (see Table S4)]. Cells were then washed three times with PBS containing 0.1% Triton X-100 and incubated for 45 min at room temperature with the secondary antibody diluted in the blocking solution. Alexa Fluor-conjugated secondary antibodies (Thermo Fisher Scientific) were used at 1/500 in 1% BSA, 0.1%Triton X-100 in PBS. Cells were then washed three times with PBS containing 0.1% Triton X-100 and were mounted for visualisation in Vectashield with DAPI (Vector Laboratories). Images were acquired with a Zeiss confocal microscope and analysed with Fiji software (Schindelin et al., 2012).

### Western blot analysis

Cell lysates were collected in Laemmli buffer [0.05 M Tris-HCl (pH 6.8), 1% SDS, 10% glycerol and 0.1% β-mercaptoethanol] and denatured for 10 min at 95°C, quantified using BCA quantification (Thermo Fisher Scientific), resolved using CriterionXT pre-cast gels (BioRad) and transferred to nitrocellulose membranes. Blocking was performed in 5% milk in TBST (TBS-Tween) buffer for 1 h at room temperature. Primary antibody incubation (p53, p21, Bcl2, Tubulin - see Table S4) was carried out overnight at 4°C in TBST containing 5% BSA. Membranes were washed three or four times in TBST at room temperature with gentle agitation and then incubated with the secondary antibody (HRP-conjugated, anti-mouse or anti-rabbit) in blocking solution (5% milk in TBS-T) at 1:5000 dilution. Membranes were then washed three or four times in TBST at room temperature with gentle agitation. Western blot quantification was performed using Fiji software version 2.0.0-rc-49/1.51d. Protein expression levels were normalized to loading control tubulin.

### RNA extraction and quantitative RT-PCR

Total RNA was extracted with the RNeasy mini kit (Qiagen) and SuperScript III reverse transcriptase (Thermo Fisher Scientific) was used for cDNA synthesis according to manufacturer's instructions. Quantitative RT-PCR was performed by amplification with SYBR Green Master Mix (Roche). The primers used are listed in Table S5. RNA samples from wild-type and mutant clones were collected from three independent experiments.

### RNA-seq analysis

Cells grown for 3 days in N2B27 media were recovered into growth media and then resuspended in RLT lysis buffer (QIAGEN). RNA extraction was performed using the QIAGEN RNeasy kit according to the manufacturer's instructions. Quality control, library preparation and sequencing were performed by the BRC Genomics Centre (Imperial College London, UK). RNA samples were quantified using a Qubit fluorometer (Thermo Fisher Scientific) and the quality assessed by TapeStation electrophoresis (Agilent). mRNA was isolated using oligo dT beads. mRNA was then fragmented, converted to cDNA and ligated to Illumina adapters. Following sample indexing, the quality of cDNA libraries was also assessed by TapeStation. Sequencing was performed using the Nextseq2000 system (Illumina). Differential gene expression analysis of the resulting sequencing data was performed by Nadia Fernandes (Imperial College London, UK) in collaboration. Sequencing reads were aligned to the mouse genome (mm9) using TopHat2 (Kim et al., 2013) and differential expression was analysed using the DESeq2 package (Love et al., 2014). The enrichment analysis for the bulk RNA-seq datasets was performed using the g:Profiler tool76. The list of upregulated, downregulated and background genes related to the differential expression analysis for the bulk RNA-seq dataset are provided in Table S1.

### Statistical methods

Statistical analysis was performed using GraphPad Prism v8.0.0 software. Statistical methods used are indicated in the relevant figure legends. No randomisation was used in experiments and operators were aware of treatment groups. Sample sizes were selected based on the observed effects and are listed in the figure legends. Statistical significance was considered with a confidence interval of 0.05%: *P<0.05; **P<0.01; ***P<0.001; ****P<0.0001.

Data obtained from cell competition assays were analysed by two-way ANOVA, followed by Holm–Sidak's multiple-comparison test. Data obtained from RT-PCR were analysed by one-way ANOVA followed by Tukey's post-hoc test. Data obtained from western blotting were analysed with an unpaired, two tailed *t*-test.

### Model simulation and approximate Bayesian inference

ODEs were solved with a custom explicit forward Euler method. Parameter distributions compatible with the model were inferred using a Markov chain Monte Carlo implemented in the multiple-try differential evolution adaptive Metropolis algorithm PyDREAM (Shockley et al., 2018). The log-likelihood function for the parameter set *θ* assumed that experimental trajectories deviations (*W*_*exp*_, *P*_*exp*_) followed a Gaussian distribution centred at each time point of the observed trajectories (*W*_*model*_, *P*_*model*_) with a width given by the square root of the population size:
(2)

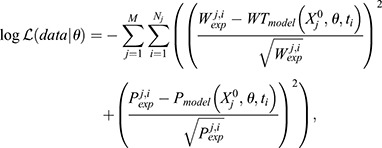
where the index *j* runs for all the experimental initial conditions 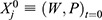, and the index *i* for all the replicates and their time points *t_i_*. Convergence of the posterior parameter distributions was assessed using the Gelman Rubin diagnostic with a threshold *R_c_*=1.2 across five different sampling chains.

### Resource competition model

The scenario in which cells compete for an external resource was modelled introducing an additional chemical resource species *R* that is depleted with cell specific rates *γ_W_* and *γ_P_*. The nutrient reduces the intrinsic apoptotic rate of each cell species *δ_W_* and *δ_P_* depending on the characteristic concentrations *r_W_* and *r_P_* to a maximum net proliferation rate *α_W_* and *α_P_*:
(3)

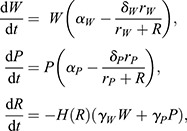
where *H*(*R*) is the Heaviside step function.

## Supplementary Material

10.1242/develop.202503_sup1 Supplementary information

Table S1. Differentially regulated genes in *p53* mutant ESCs compared to wild-type cells at day 3 of differentiation.

Table S2. Cell numbers and growth rates of wild-type and *p53* mutant cells between days 0 to day 4 of differentiation at the different cell densities platted. The rate of elimination of wild-type cells when co-cultured with *p53* mutant cells is also indicated.

Table S3. Proportion of wild-type present between days 1 and 4 of differentiation when co-cultured with different proportions of *p53* mutant cells. The rate of elimination of wild-type cells is also indicated.

Table S4. Antibodies
