# Supplementary material for: Mutation of p53 increases the competitive ability of pluripotent stem cells
Source: Development. 2024 Jan 19;151(2):dev202503. doi: 10.1242/dev.202503 (PMC10820806; doi:10.1242/dev.202503)
Supplement: Supplementary information [file develop-151-202503-s1.pdf]

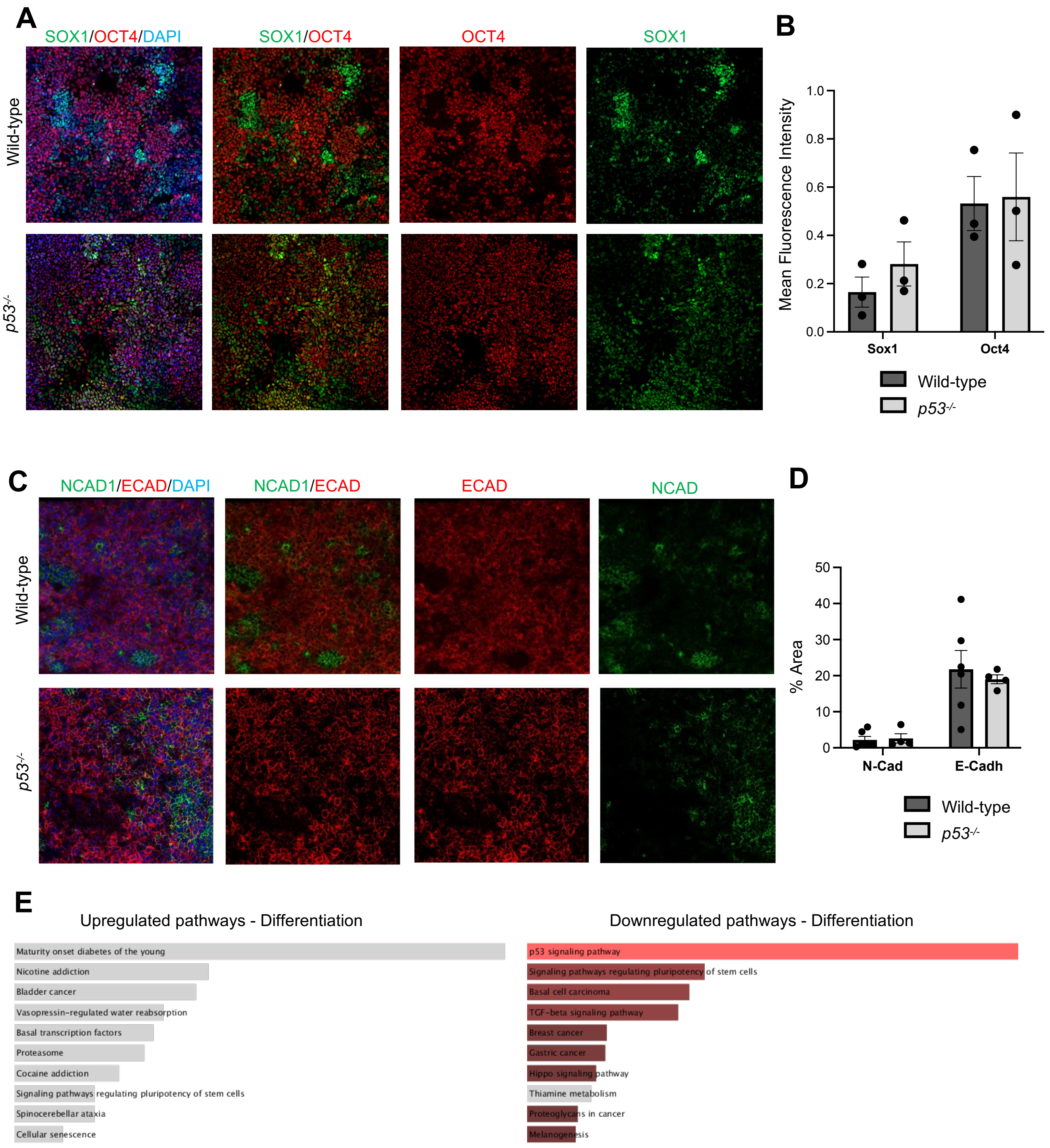

Fig. S1. Differentiation ability of *p53* mutant cells. A. Immunostainings for SOX1 and OCT4 in wild-type and *p53*$^{-/-}$ cells cultured separately for 3 days in N2B27. **B.** Quantification of a. **C.** Immunostainings for ECADHERIn and NCADHERIN in wild-type and *p53*$^{-/-}$ cells cultured separately for 3 days in N2B27. **D.** Quantification of b. **E.** Upregulated and downregulated pathways in *p53*$^{-/-}$ cells compared to wild-type cells by RNAseq. Data were obtained from three independent experiments and are shown as the mean + SEM (**A-D**).

Development • Supplementary information

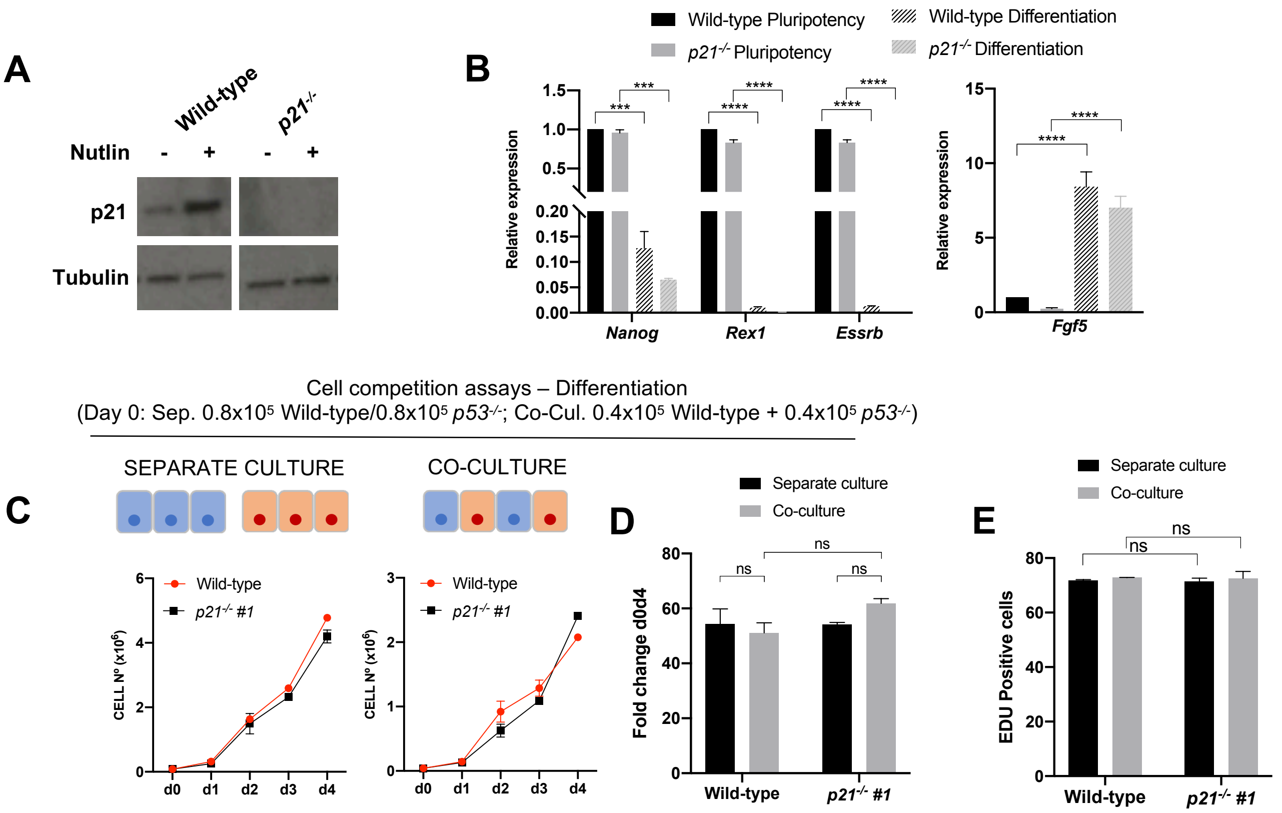

Fig. S2. Competitive ability of *p21* mutant cells. A. P21 levels in wild-type and *p21*$^{-/-}$ clones untreated or treated with the P53 activator Nutlin-3a for 4h. **B.** Quantitative RT-PCR showing gene expression levels of naïve and primed pluripotency markers in wild-type and *p21*$^{-/-}$ ESCs in pluripotency and differentiation culture conditions. Gene expression is normalized against beta-Actin. **C.** Growth curves of wild-type and *p21*$^{-/-}$ cells cultured for 4 days in separate or co-culture differentiation conditions. **D.** Fold change in wild-type and *p21*$^{-/-}$ cell numbers between day 0 and day 4 when cultured separately or co-cultured. **E.** Percentage of EDU incorporation in wild-type and *p21*$^{-/-}$ cells at day 4 cultured separately or co-cultured in differentiation conditions. Data were obtained from three independent experiments and are shown as the mean + SEM (**B-E**).

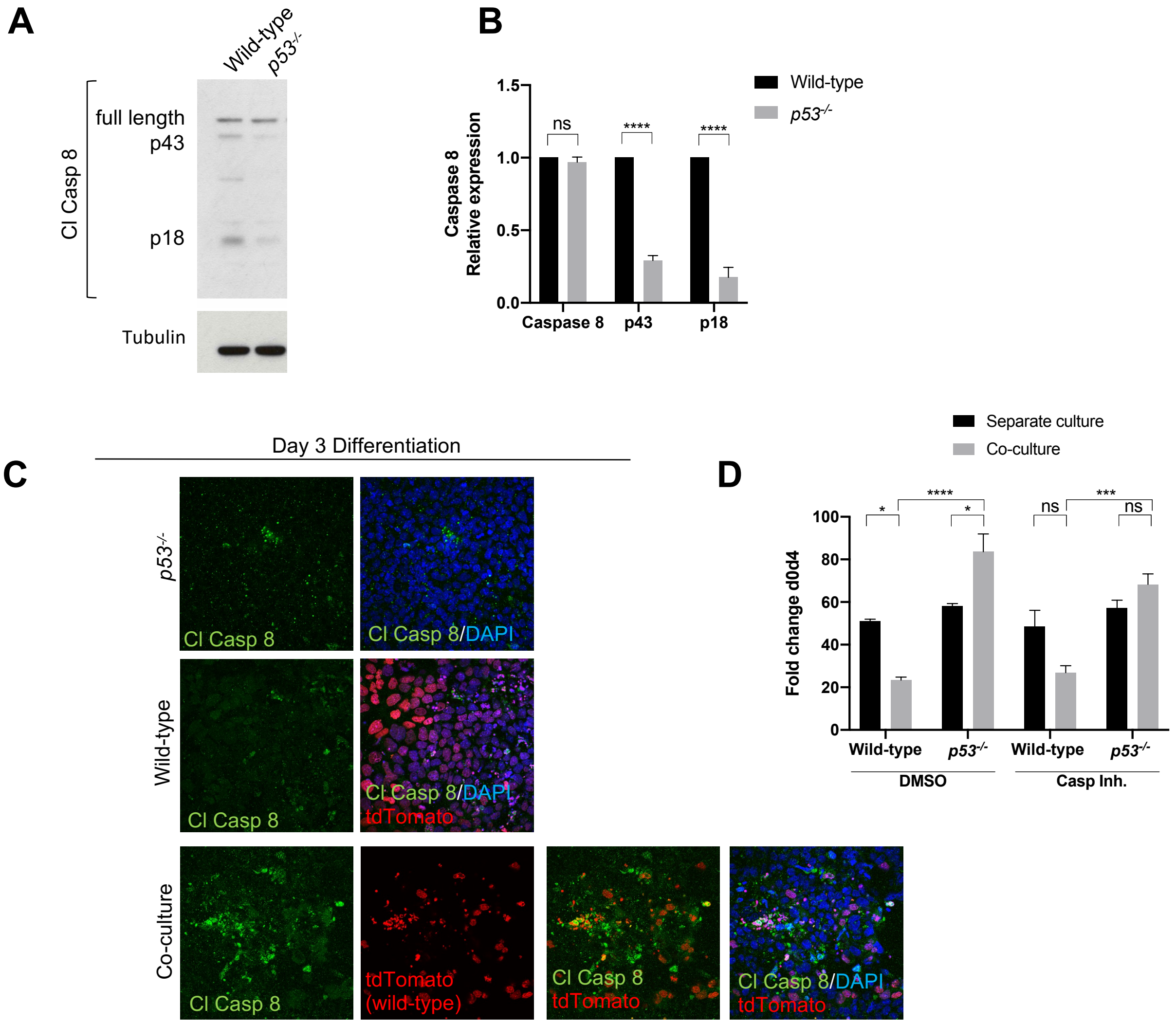

Fig. S3. Apoptosis levels in wild-type and *p53* mutant cells.

A. Levels of un-cleaved Caspase 8 and its cleaved forms p41 and p18 in wild-type and *p53*$^{-/-}$ cells cultured separately for 3 days in differentiation conditions. **B.** Quantification of a. **C.** Immunostainings of wild-type and *p53*$^{-/-}$ cells cultured in separate conditions or co-cultured for 3 days showing an increase in Cleaved Caspase 8 in wild-type cells. **D** Fold change in wild-type and *p53*$^{-/-}$ cell numbers between day 0 and day 4 when cultured in separate conditions or co-cultured in differentiation conditions and treated with DMSO or pan caspase inhibitors from day 3 to day 4. Data were obtained from three independent experiments and are shown as the mean + SEM (**A**-**D**).

Development • Supplementary information

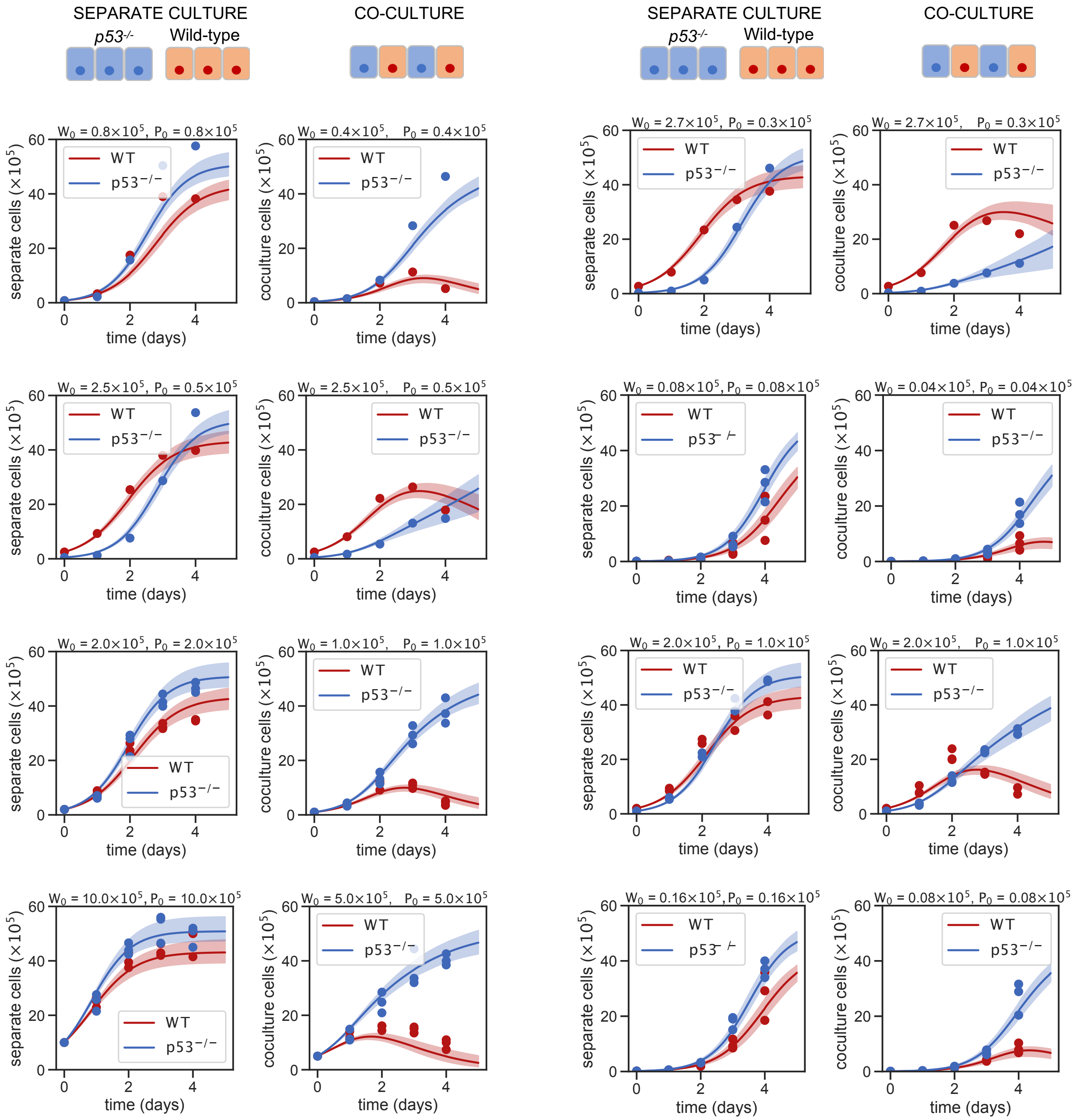

Fig. S4. Testing the mathematical model for cell competition. Comparison of the experimental data (one circle per replicate) with the model prediction (lines) for the direct competition model (Eq. 1) for all the different 24 experimental plating conditions used in the inference. Shaded zones show model prediction for the inferred parameter region with likelihood >90% of its maximum. Parameter inference values can be found in Fig. S4.

Development • Supplementary information

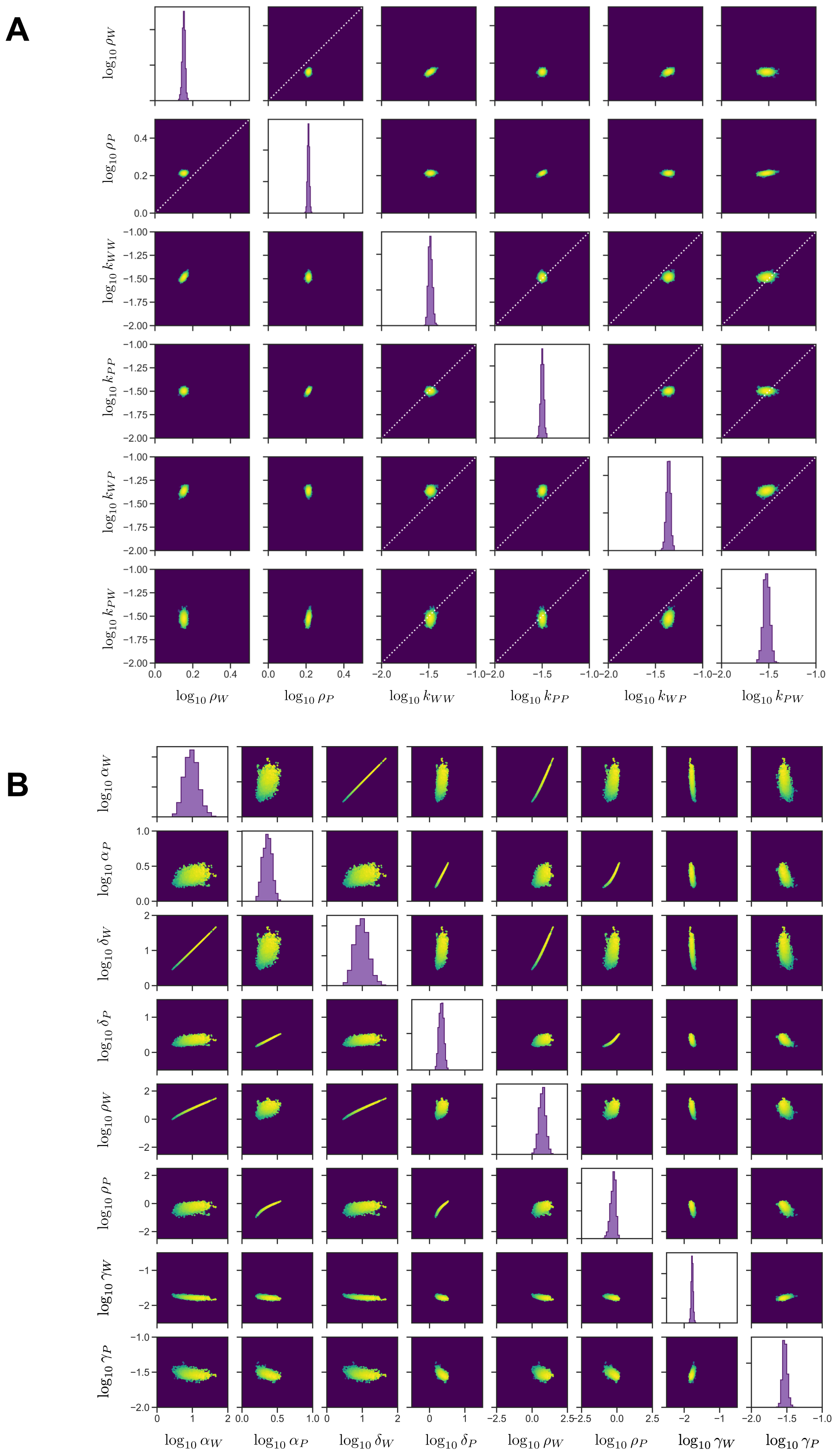

Fig. S5. Posterior credibility distributions for the direct competition model (Eqn 1). Posterior credibility distributions for the direct competition model (Eq. 1) (**A**), and for the resource competition model (**B**). Diagonal panels show marginal posterior distribution histograms. Off-diagonal panels show the sampled pairwise joint distribution for all the possible parameter pairs. Colour indicates magnitude of the likelihood.

Development • Supplementary information

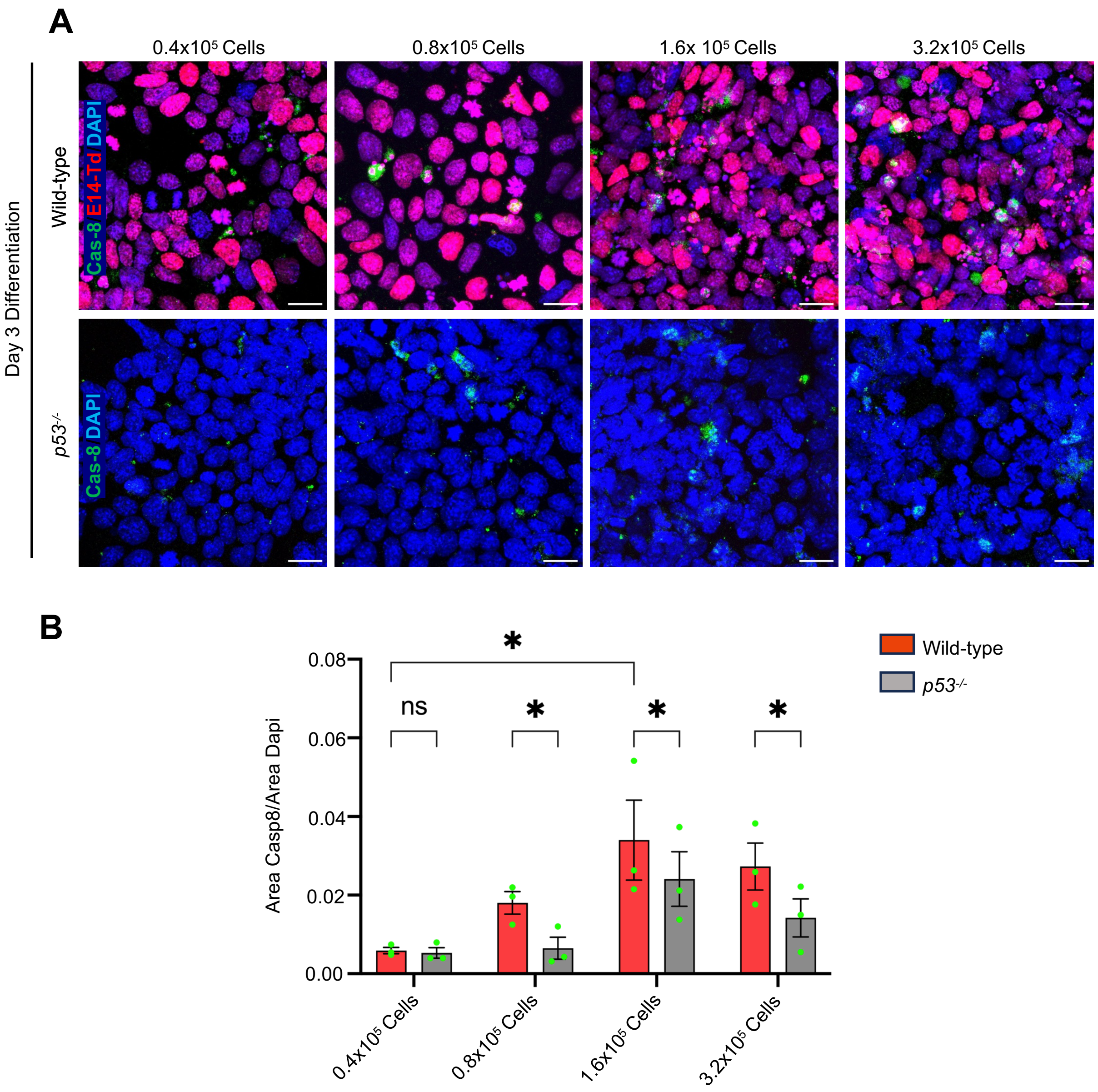

Fig. S6. Caspase 8 levels during cell competition. A. Expression of Caspase 8 in wild-type and *p53*$^{-/-}$ cells cultured for 3 days in separate conditions with 0.4x10^{5}, 0.8x10^{5}, 1.6x10^{5} and 3.2x10^{5} cells seeded per well at day 0. **B.** Quantification of A. Data were obtained from three independent experiments and are shown as the mean + SEM (**A-C**).

Development • Supplementary information

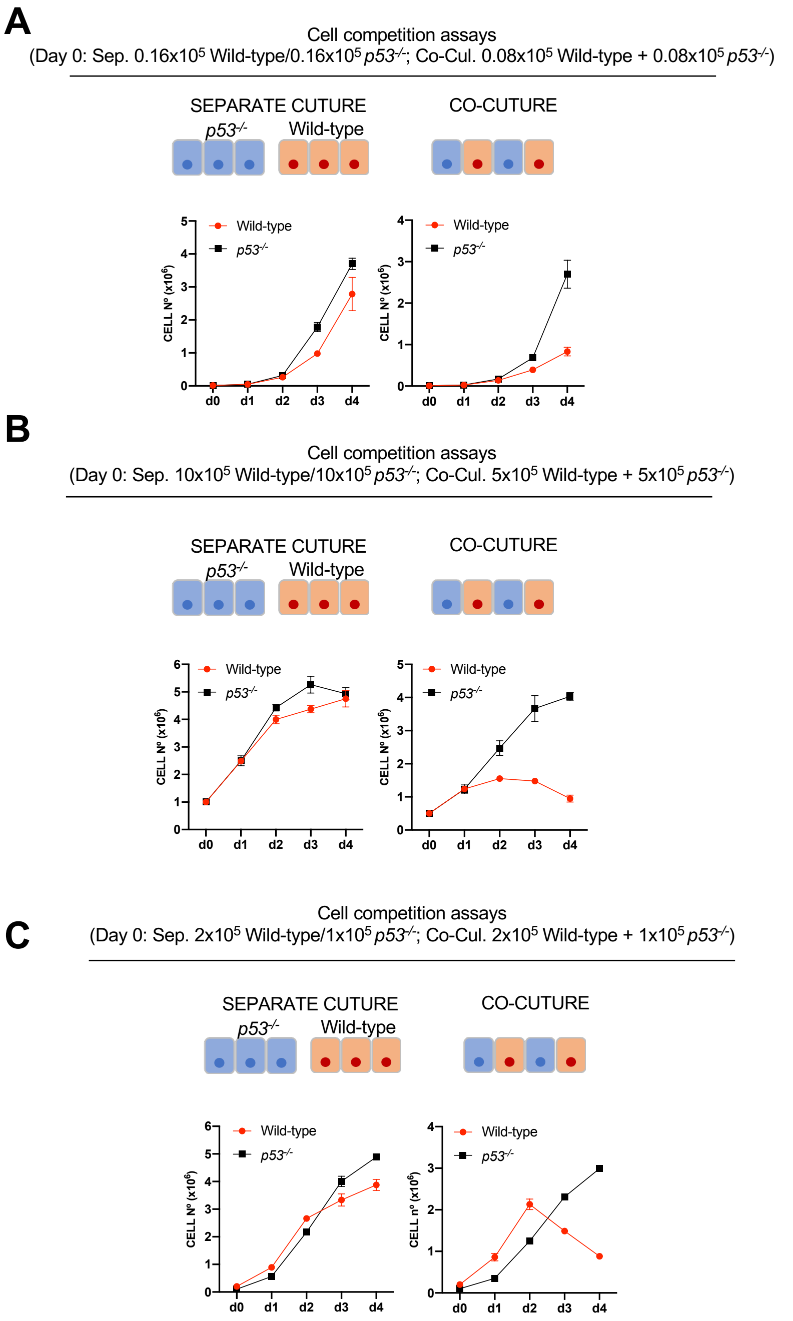

Fig. S7. Cell competition effects at different plating densities. A. Growth curves of wild-type and *p53*$^{-/-}$ cells cultured for 4 days in separate or co-culture conditions with a starting cell number of 0.016x10^{6} cells seeded per well. **B.** Growth curves of wild-type and *p53*$^{-/-}$ cells cultured for 4 days in separate or co-culture conditions with a starting cell number of 1x10^{6} cells seeded per well. **C.** Growth curves of wild-type and *p53*$^{-/-}$ cells cultured for 4 days in separate or co-culture conditions with a starting cell number of 0.2x10^{5} wild-type cells and 0.1x10^{5} *p53*$^{-/-}$ cells seeded per well. Data were obtained from three independent experiments and are shown as the mean + SEM (**A**-**C**).

Development • Supplementary information

Table S1. Differentially regulated genes in *p53* mutant ESCs compared to wild-type cells at day 3 of differentiation.

Available for download at
https://journals.biologists.com/dev/article-lookup/doi/10.1242/dev.202503#supplementary-data

Table S2. Cell numbers and growth rates of wild-type and *p53* mutant cells between days 0 to day 4 of differentiation at the different cell densities platted. The rate of elimination of wild-type cells when co-cultured with *p53* mutant cells is also indicated.

Available for download at
https://journals.biologists.com/dev/article-lookup/doi/10.1242/dev.202503#supplementary-data

Table S3. Proportion of wild-type present between days 1 and 4 of differentiation when co-cultured with different proportions of *p53* mutant cells. The rate of elimination of wild-type cells is also indicated.

Available for download at
https://journals.biologists.com/dev/article-lookup/doi/10.1242/dev.202503#supplementary-data

Table S4. Antibodies

Available for download at
https://journals.biologists.com/dev/article-lookup/doi/10.1242/dev.202503#supplementary-data

Table S5. Primers used in quantitative RT-PCR

Gene	Forward	Reverse
Gapdh	5’ CATGGCCTTCCGTGTTCCTA 3’	5’ GCGGCACGTCAGATCCA 3’
Fgf5	5’ AAAGTCAATGGCTCCCACGAA 3’	5’ CTTCAGTCTGTACTTCACTGG 3’
Esrrb	5’ GGACACACTGCTTTGAAGCA 3’	5’ ACAGATGTCTCTCATCTGGC 3’
Nanog	5’ CTTACAAGGGTCTGCTACTGAGATGC 3’	5’ TGCTTCCTGGCAAGGACCTT 3’
Rex1	5’ CGAGTGGCAGTTTCTTCTTGG 3’	5’ GACTCACTTCCAGGGGGCAC 3’

Development • Supplementary information
