# Supplementary material for: Mutation of p53 increases the competitive ability of pluripotent stem cells
Source: Development. 2024 Jan 19;151(2):dev202503. doi: 10.1242/dev.202503 (PMC10820806; doi:10.1242/dev.202503)
Supplement: Supplementary file 1 [file develop-151-202503_review_history.pdf]

Mutation of p53 increases the competitive ability of pluripotent stem cells

Salvador Perez Montero, Pranab K. Paul, Aida di Gregorio, Sarah Bowling, Solomon Shepherd, Nadia J. Fernandes, Ana Lima, Ruben Pérez-Carrasco and Tristan A. Rodriguez
DOI: 10.1242/dev.202503

Editor: Patrick Tam

Review timeline
Original submission: 28 February 2022
Editorial decision: 28 March 2022
Resubmission: 7 November 2023
Editorial decision: 4 December 2023
First revision received: 15 December 2023
Accepted: 18 December 2023

Original submission

First decision letter

MS ID#: DEVELOP/2022/200705

MS TITLE: Levels of p53 expression determine the competitive ability of embryonic stem cells during the onset of differentiation.

AUTHORS: Salvador Perez Montero, Sarah Bowling, Ruben Perez-Carrasco, and Tristan A Rodriguez

I have now received all the referees' reports on the above manuscript, and have reached a decision. The referees' comments are appended below, or you can access them online: please go to BenchPress and click on the 'Manuscripts with Decisions' queue in the Author Area.

As you will see, the referees express considerable interest in your work, but have some significant criticisms and recommend a substantial revision of your manuscript before we can consider publication. If you are able to revise the manuscript along the lines suggested, which may involve further experiments, I will be happy receive a revised version of the manuscript. Your revised paper will be re-reviewed by one or more of the original referees, and acceptance of your manuscript will depend on your addressing satisfactorily the reviewers' major concerns. Please also note that Development will normally permit only one round of major revision. If it would be helpful, you are welcome to contact us to discuss your revision in greater detail. Please send us a point-by-point response indicating your plans for addressing the referee's comments, and we will look over this and provide further guidance.

Please attend to all of the reviewers' comments and ensure that you clearly highlight all changes made in the revised manuscript. Please avoid using 'Tracked changes' in Word files as these are lost in PDF conversion. I should be grateful if you would also provide a point-by-point response detailing how you have dealt with the points raised by the reviewers in the 'Response to Reviewers' box. If you do not agree with any of their criticisms or suggestions please explain clearly why this is so.

Reviewer 1

© 2023. Published by The Company of Biologists under the terms of the Creative Commons Attribution License (https://creativecommons.org/licenses/by/4.0/).

Advance summary and potential significance to field

p53 is suggested to be involved in the elimination of loser cells in several cell competition systems. This paper describes a novel finding that p53-mutant cells behave as super-competitors that eliminate wild-type cells from cell competition during the onset of differentiation of ES cells. The authors also showed, by combining mathematical modeling and cell culture experiments, that elimination of wild-type cells by p53 mutant cells is mediated by short-range interactions, not by deprivation of nutrients or mechanical stresses. Finally, the authors showed that changes in the local cell neighborhood correlate with changes in cell number and concluded that the relative number of neighboring cells with different competitive abilities influences the replacement of wild-type cells. These findings are important for the field of cell competition and developmental biology because they add a new distinct mode of cell competition during ES cell differentiation and a novel role of p53 in cell competition.
However, the data supporting this last point were not convincing.

Comments for the author

Major comments:

1. The title does not present the conclusion of this paper. In this study, the authors demonstrated that p53 mutant cells behave as super-competitors, but they did not explore the relationship between the levels of p53 expression and the competitive activities of ES cells. Therefore, it is not appropriate to conclude that p53 expression levels determine the competitive activity of the cells. Although this could be mentioned in the discussion, it could not be a conclusion and/or title unless additional experiments were performed that directly compared competitive activities of the cells with different levels of p53 expression, such as p53+/-, Mdm4+/-, p53-/-, and wild-type ES cells.
2. In Figures 5 and S5, the authors examined changes in the ratio of neighboring cell types during cell competition. By showing the correlations between the ratios of neighboring cells and growth curves, the authors concluded that the relative number of neighboring p53-/- cells is a factor affecting the elimination of wild-type cells. Although this is an intriguing interpretation, it is unclear whether the observed changes in the neighborhood are the causes or consequences of cell competition. Therefore, this point needs to be clarified. For example, as shown in Figure 1D, strong elimination of wild-type cells occurred between days 2 and 3. Because elimination of wild-type cells promotes replacement of the neighboring cells, it is likely that elimination of wild-type cells caused an increase in the relative numbers of p53 -/- neighbors between days 2 and 3 (Figure 5B). According to this interpretation, changes in the neighborhood are the consequences of cell competition. If the ratio of neighboring p53-/- cells is important for elimination, then a stronger elimination of wild-type cells should be observed between days 3 and 4. However, it was difficult to estimate the elimination rate from the growth curve. Is it possible to quantify the cell elimination rates from the growth curve and correlate them with the ratio of neighborhood cell types? If the ratio of neighboring cell types is an important factor triggering elimination, then the increase in the ratio of p53-/- neighbors should precede the increase in elimination of wild-type cells.

Related to the above point, the weakness of the current analyses lies in the comparison of cell neighborhoods with growth curves. Because growth curves lack spatial information and do not indicate which cell will be eliminated, it is difficult to correlate the ratio of the cells' neighbors with the cells' fates. Analyses of the correlation between the ratio of neighboring p53-/- cells and the ratio of cells expressing apoptotic markers, such as cleaved caspases-3 and -8, would be a more direct experiment addressing the importance of the ratio of p53-/- cells in the neighborhood for cell fate.

In the case of co-culture with Bcl2^In cells (Figure 5D), Bcl2^In cells were not eliminated, and the ratio of the neighboring p53-/- cells was unchanged. However, no change in the neighborhood does not necessarily imply that the cell neighborhood plays a role in cell competition. If the cell neighborhood plays a role in this co-culture, then a forced increase in the ratio of p53-/- neighbors by strongly reducing the number of initial Bcl2^In cells (and also increasing the total number of plating cells) should turn Bcl2^In cells into losers. Do the authors expect this to occur?

Minor comments:

1. Lines 77-79. Please check whether “8 x 10^5 cells” and “4 x 10^5 cells” are typos of “0.8 x 10^5 cells” and “0.4 x 10^5 cells”, respectively.

© 2023. Published by The Company of Biologists under the terms of the Creative Commons Attribution License (https://creativecommons.org/licenses/by/4.0/).

2. Line 130. “(1)” may be better described by “Eq. (1)” because the equation is referred to as “Eq. (1)” in Line 201.
3. Lines 231-232. The description “cells grown on the outside layer grew slower than those cultured on the inside” does not match with the data of Figure 4G. Please check this conclusion drawn from the data.
4. Lines 301-305. Please cite the authors’ previous paper(s) for Bowling et al., 2018.

Reviewer 2

Advance summary and potential significance to field

In this article, Montero et al explore the hypothesis that cell competition during embryonic development can be dictated by differences in the levels of p53 expression between neighbour cells. To test this idea, the authors perform a series of quantitative analyses comparing the proliferative capacity of WT and p53 null mouse embryonic stem cells in various culture / co-culture setups, using notably mathematical modelling to inform their interpretation of the data. They find that absence of p53 can turn cells into super-competitors that eliminate their wild-type neighbours through the direct induction of apoptosis. They also find that this process is initiated upon cell differentiation and happens at a short range, most likely through direct cell-cell contact.

In my opinion, this is a very interesting report. I do not see any clear flaw in the reasoning of the authors, experiments are well conducted and generally convincing, the computational model is helpful to distinguish the respective influence of intrinsic growth differential versus cell competition on the population dynamic. I think this article could be a great addition to the literature on cell competition during development and will prompt further research on the underlying molecular mechanisms in the future.

Comments for the author

I outline below some suggestions to strengthen some of the results presented:
Comments on the data presented
Do p53-/- cells differentiate at the same rate as WT cells (Fig1)
In fig.1 the authors observe that cell competition is most apparent upon culture in N2B27 and relatively negligible in pluripotency conditions. Line 98, the authors claim that “this behaviour was not due to the inability of p53 mutant cells to initiate differentiation”. This is an important point. When we compare Fig1 C and E, we observe that p53-/- cells proliferate faster during differentiation than during pluripotency whereas WT cells keep the same proliferation rate in both conditions in separate cultures. In Fig 1F we also see that Nanog in p53-/- is not downregulated as much as in WT cells and consistently, FGF5 is lower in p53-/- cells than in WT cells.
These data indicate that p53-/- cells respond differently to the differentiation medium compared to WT cells. Therefore, there is a possibility that N2B27 induces a more heterogeneous differentiation in p53-/- cells or that p53-/- cells remain stuck in an intermediate differentiation stage that is more proliferative. If this is the case, one could argue that the competition behaviour inferred throughout the article is not a direct consequence of p53 sensing but instead an indirect effect of p53 KO promoting “winner” cell subpopulations in the culture.
To address this, the authors could stain for a few markers such as Oct4, SOX1, ECAD and NCAD and assess how efficient or homogeneous p53-/- differentiation is compared to the WT situation.
Is WT cell elimination contact dependent (Fig4F-I)
The “fences” experiment is very elegant but I think that some clarification could be added and that a simple additional experiment could strengthen the data:
1) I am surprised to see that the cells are growing faster when grown in the inner region, could the authors comment on the possible reasons for this observation?
2) The description of the experiment in the text is clear but I think that at least a bright field image of the culture would be helpful to illustrate the experimental setup and show the actual distance that separate the two population during the experiment.
3) To strengthen the idea that WT cell elimination requires short range signalling or cell contact, the authors could let the cells grow a sufficient amount of time in order for the 2 populations to reach one another and then quantify the level of cleaved caspase 8 at the front of encounter versus away from that front.

© 2023. Published by The Company of Biologists under the terms of the Creative Commons Attribution License (https://creativecommons.org/licenses/by/4.0/).

Ideally this would be done in a different “fence format”, for example using a removable 3-well insert as those provided by Ibidi for wound healing assays. WT cells would be seeded on wells 1 and 2 and p53-/- cells on well 3. Comparing the WT/WT interface vs the WT/p53-/- interface could provide strong evidence that contact is required for WT cell elimination by p53-/- cells.
Neighbourhood analysis (Fig5)
The conclusion from the neighbourhood analysis shown in Fig 5A-D is not as intuitive as it is presented by the authors Line 269 “These results are consistent with cell neighbourhood having a role in the outcome of competition”
For example, could these data be explained by differential growth rate only? Given that the net growth of p53-/- cells is much higher than WT cells in co-culture, we could reasonably expect that WT cells become progressively surrounded by p53-/- cells as p53-/- cells become the dominant population in the culture and this, regardless of whether neighbourhood plays a role or not (same for Bcl2 without Dox). Note that the Bcl2 rescue on the neighbourhood profile is interesting but Bcl2 also rescues the growth rate of the population.
Conversely, if we follow the author’s reasoning and assume that WT cells die when surrounded by p53-/- cells, shouldn't we expect surviving cells having mostly WT neighbours in the end (in contrast to what we see in the data)?
I think it is not easy to mentally disentangle the contribution of these respective “forces” that dictate the neighbourhood profile. To address this, perhaps the authors could build on their existing mathematical model to predict the evolution of neighbourhood profiles in different scenarios and compare the results to their experimental data.
If this is not feasible at this point (I appreciate the model does not currently take into consideration any spatial element), an alternative idea could be to stain for cleaved Caspase 8 at day3 or 4 and show that Caspase 8 negative WT cells tend to be surrounded by p53-/- cells whereas Caspase8 positive WT cells have a higher number of p53-/- cells.
Minor comments:
* Line 88: I think a diagram explaining the strategy and the validation of the p53-/- cell line should be included in the remanuscript (as a supplementary figure).
* Line 145 One of the symbol in the equation is not defined.
* Line 145 Fig3A-B should be Fig 2.A-B
* Line 219 Eq (3) is not indicated in the Materials and Methods
* Fig. 4B For consistency, it would be good if the y axis could show “d0d4 ratio” as in other bar plots.
* Line 234 – 237: “the fact that the number of cells in the culture dish can double without increasing the degree of wild-type apoptosis, also suggests that this elimination is not due to mechanical stress, as has been shown to be the case for MDCK cells with increased p53 expression” Here I assume that the assumption is that the cells are becoming more packed, hence the idea of mechanical stress. This could be more explicit, please clarify.
Fig 4G is a bit misleading, at first it looks like the cells in the inner region proliferate slower than the cells in the outer region, while the opposite is true (based on Fig4. H). The graph could perhaps be represented with log scale for cell number or as time versus relative number of cells (cell number / initial number)
Fig. 5 – It can take some time to understand the histograms shown in Fig 5. It would be useful to add a label for the x axis. Maybe “% of WT cells”. I did not understand why a + and – sign was shown in the graduation of the x axis. The y axis label is also confusing (removing the % sign could help).
Fig. 5 E The results of the neighbour analysis at low density are clearly in favour of the author’s thesis but to be even more convincing, I think the authors should show a comparison of the values of the growth rates shown in S5 A and B (to show that indeed, WT cells at low confluency are proliferating at their normal rate).

Reviewer 3

Advance summary and potential significance to field

The manuscript from the Tristan Rodriguez group follows on a growing body of work, including their own, studying mechanisms of cell competition in mouse ES cells. In particular, they seek to understand the mechanisms by which p53 mutant cells out-compete wild-type cells – a phenomenon previously observed in vitro and in vivo in mouse embryos and interspecies chimeras. Working with mouse ES cells in vitro, they use a combination of mathematical modelling and cell-based assays to

© 2023. Published by The Company of Biologists under the terms of the Creative Commons Attribution License (https://creativecommons.org/licenses/by/4.0/).

characterize the cellular and molecular mechanisms that underlies this observation. Their overall conclusion is that short-range signalling (ie. Direct cell-cell contact) between wt and P53 mutant cells induces wt cell apoptosis, and that this does not occur by virtue of any competition for nutrients or space. Intriguingly, the competition is context dependent - only upon exit from pluripotency does competition ensue.

There are some intriguing pieces of data in the manuscript, and the scope of their questions regarding the role of P53 and cell competition will certainly be of interest to the Development readership. A majority of the cell competition scenarios described in mammals seem to converge on P53, and thus understanding the mechanism by which P53 mutant cells super-compete could be generalizable across many other biological contexts.

Comments for the author

In its current state, the manuscript is a little bit thin on new, concrete biological insight since it is already well established that embryonic p53 mutant cells outcompete wild-type cells (Dejosez et al, Science, 2013; Zheng et al, Nature, 2021). Moreover, the role of cell-cell contact in mediating cell competition is also described in several systems including mouse ES cells (Diaz-Diaz et al, Dev Cell, 2017; Levayer et al, Nature 2015). As such, to merit publication in Development the mechanistic understanding of P53-mediated super-competition would need to be more solidly explored than is currently the case in this version of the manuscript. To this end, there is a large degree of over- and/or mis-interpretation of data in their conclusions, as well as several essential controls that are missing. These issues would have to be thoroughly addressed before publication in the journal. However, I think if they can flesh out some of their observations more thoroughly (as outlined in my specific points below) then they can sufficiently overcome this criticism. In this case, the manuscript would become an interesting addition to the cell competition oeuvre, and worthy of publication in Development.
Major Points:
1. Controls for CRISPR knockout: There are several essential controls missing. Generation/Use of the p53 CRISPR knock-out line is inadequately described. Are the “wild-type” cells isogenic controls? The methods and/or the text should clearly state what they are considering WT cells for these experiments. I also have concerns about off-target effects. There are no controls included to rule this out. At least some data with a second knock-out line should be shown before publication (and/or a rescue experiment would also be nice). Otherwise how do we know anything they show is specific for P53?
2. Ruling out alternative models: It is very interesting that robust induction of cell competition only happens once cells exit from pluripotency. This also fits nicely with the timing of cell elimination events that the same group observes in vivo in the post-implantation mouse embryo. How ES cells are able to suppress cell competition in one state, but not another is a fascinating question. However, the relationship between differentiation and competition is not satisfactorily explored. While I fully appreciate that solving the potential role of P53 in transcriptional rewiring during the onset of differentiation is beyond the scope of this paper, I think the conclusions the authors make against this possibility are too strong. Is it not possible that subtle, heterogeneous differences in differentiation dynamics may also contribute to competitive outcomes? Simply exploring this with qPCR is not sufficient to rule this out. Moreover, although the data in Figure 1F shows similar trends between WT and P53 mutant cells, the magnitude is very different and the kinetics are also likely to be quite different (but this isn’t explored). Ideally, more data should be added to address this, and at minimum, the conclusions should be softened. As discussed in point 3 below, subtle differences in differentiation dynamics could potentially be another contributing factor to explain why the caspase stainings/inhibitor data doesn’t seem to completely account for the overall growth disadvantage of WT cells.
3. Apoptosis data is not convincing: Although the Bcl2-induction experiments are very nice, the Caspase inhibitor data in Figure S2, together with the Cas-3/8 quantification shown in Figure 1H and Figure S2 doesn’t sufficiently support the conclusion apoptosis is sufficient to explain the competitive loss of WT cells. The authors concede in the text that there might be something else going on but do not satisfactorily investigate these possibilities. As they mention, there may be caspase-independent death happening. There also may be live cell extrusion events without any caspase activation. Both of these possibilities may perhaps be addressed with live imaging experiments. Cell death is notoriously challenging to capture in IF – Casp3+ dying cells may be washed away and/or the time window of Casp3+ may be missed. Live imaging would allow them to

© 2023. Published by The Company of Biologists under the terms of the Creative Commons Attribution License (https://creativecommons.org/licenses/by/4.0/).

get around these challenges and enable quantification of a wider diversity elimination events rather than just caspase-dependent apoptosis-per se. I am also wondering about senescence? Is it possible that WT cells instead enter a senescent state? Although perhaps less likely to be rescued by Bcl2 over-expression, wt cells becoming increasingly senescent would be somewhat in line with what has been observed in the hematopoietic system by the Medzhitov lab. This could be addressed with a quick IF staining.

4. Ruling out Mechanical Competition: I don't think the authors can sufficiently rule this out and I don't fully understand their rationale for doing so based solely on the data from the perfusion experiments. Perhaps one source of my confusion is that there is plenty of quantification of cell number, but no discussion or quantification of confluence or of cell density. In fact, the neighbourhood data in Figure 5 also suggests that cell density could play a role. This should be explored.

5. Mathematical Model Confusion: this reviewer is not a mathematician but I am a biologist with great appreciation for cross-disciplinary approaches. I tried my best to make sense of the data from the model but I had a pretty hard time ascertainingthe parameters and the parameter space explored. I understand the value of the conclusions but how we got there is a little bit opaque from my perspective and I suspect I would not be alone on this amongst the Development readership. In more concrete terms what is entailed in the competition "strength" parameter k? The manuscript cites a prior study of bacterial population dynamics where a similar model was previously used, but it would help to more explicitly explain this if possible. I also struggled with Figure 2D which is of course absolutely critical to understand the parameters that actually fit the experimental data and (I think?) encapsulates the results that are the key utility of the mathematical model. If the authors could come up with a complimentary way to convey which part of the parameter space the experimental data occupies, this would be very helpful. I suspect that the data shown in Figure S4 partly addresses this comment but I am not at all equipped to understand what is shown in that Figure – this could also be much better explained than it currently is. Perhaps another schematic (similar to Figure 2A, B which are fantastic) would also be useful? Or could the inferred parameters somehow be indicated on that pre-existing schematic? As I read on, the data shown in Figure 3I was immensely helpful to clarify for me how the model was working – ie. examples of how changing the parameters can fit different experimental conditions. In my view, it may be worth considering restructuring the paper somewhat so that this data can go together with the initial description of the model. I think the fact that until then we only see modelling data from one set of parameter values makes it a bit challenging to appreciate and understand the range of possibilities and the potential predictive power of the model.

Minor Points:

-figure call-outs are not always correct,-I assume the TdTomato cells in Fig 5 are the WT cells but this isn't explicitly labelled anywhere on the figure or in the figure legend/text (unless I missed it)

First revision

Author response to reviewers' comments

Response to the Reviewers

We are grateful for the constructive and supportive comments of the reviewers that have helped us improve our manuscript. We detail below our responses to the specific points that they raised and indicate what changes to the manuscript have been performed.

Reviewer #1

Major Points:

1. The title does not present the conclusion of this paper. In this study, the authors demonstrated that p53 mutant cells behave as super-competitors, but they did not explore the relationship between the levels of p53 expression and the competitive activities of ES cells. Therefore, it is not appropriate to conclude that p53 expression levels determine the competitive activity of the cells. Although this could be mentioned in the discussion, it could not be a conclusion and/or title unless additional experiments were performed that directly compared competitive activities of the cells with different levels of p53 expression, such as p53+/-,

© 2023. Published by The Company of Biologists under the terms of the Creative Commons Attribution License (https://creativecommons.org/licenses/by/4.0/).

Mdm4+/-, p53-/-, and wild-type ES cells.

Following the reviewer's suggestion, we have changed the title to "Mutation of p53 increases the competitive ability of pluripotent stem cells".

2. In Figures 5 and S5, the authors examined changes in the ratio of neighboring cell types during cell competition. By showing the correlations between the ratios of neighboring cells and growth curves, the authors concluded that the relative number of neighboring p53-/- cells is a factor affecting the elimination of wild-type cells. Although this is an intriguing interpretation, it is unclear whether the observed changes in the neighborhood are the causes or consequences of cell competition. Therefore, this point needs to be clarified. For example, as shown in Figure 1D, strong elimination of wild-type cells occurred between days 2 and 3. Because elimination of wild-type cells promotes replacement of the neighboring cells, it is likely that elimination of wild-type cells caused an increase in the relative numbers of p53 -/- neighbors between days 2 and 3 (Figure 5B). According to this interpretation, changes in the neighborhood are the consequences of cell competition. If the ratio of neighboring p53-/- cells is important for elimination, then a stronger elimination of wild-type cells should be observed between days 3 and 4. However, it was difficult to estimate the elimination rate from the growth curve. Is it possible to quantify the cell elimination rates from the growth curve and correlate them with the ratio of neighborhood cell types? If the ratio of neighboring cell types is an important factor triggering elimination, then the increase in the ratio of p53-/- neighbors should precede the increase in elimination of wild-type cells.

To address this point, we have done several things. First, we provide in Supplementary Table 2 the rate of wild-type elimination for the different densities of cells plated in Figures 2 and S4. Second, to further test the importance of the relative number of neighbours for wild-type cell elimination, we have plated cell competition experiments with the following ratios of wild-type to *p53*$^{-/-}$ ratios: 1:1; 1:3; 1:5 and 1:10. We then calculated the rate of elimination per day and between condition. When this is done, we observe that as expected, the rate of loser cell elimination increases with time and is highest between days 3 and 4 of coculture. Furthermore, we also observe that as the proportion of wild-type cells is diluted, the rate of elimination increases. This corelation is apparent right from between day 1 and 2 and suggests that a higher proportion of winner neighbours accelerates the elimination of losers. This data supports that the hypothesis that the relative neighbourhood of loser cells is factor regulating their elimination and is now presented in Supplementary Table 3 and is discussed in lines 306-314 of the text.

Related to the above point, the weakness of the current analyses lies in the comparison of cell neighborhoods with growth curves. Because growth curves lack spatial information and do not indicate which cell will be eliminated, it is difficult to correlate the ratio of the cells' neighbors with the cells' fates. Analyses of the correlation between the ratio of neighboring p53-/- cells and the ratio of cells expressing apoptotic markers, such as cleaved caspases-3 and -8, would be a more direct experiment addressing the importance of the ratio of p53-/- cells in the neighborhood for cell fate. In the case of co-culture with Bcl2^In cells (Figure 5D), Bcl2^In cells were not eliminated, and the ratio of the neighboring p53-/- cells was unchanged. However, no change in the neighborhood does not necessarily imply that the cell neighborhood plays a role in cell competition. If the cell neighborhood plays a role in this co-culture, then a forced increase in the ratio of p53-/- neighbors by strongly reducing the number of initial Bcl2^In cells (and also increasing the total number of plating cells) should turn Bcl2^In cells into losers. Do the authors expect this to occur?

We have performed cleaved caspase 3 staining and analysed the distribution of dead cells relative to their genotype. However, no clear pattern could be observed as it was hard to distinguish apoptotic signals in dying cells from apoptotic signals in engulfed cells. To overcome this problem, and test the importance of neighbourhood, we have used culture wells from Ibidi, where the two wells are separated by a silicone insert that creates a defined cell free gap. Using this system winner and loser cells come together, but no straight boundary in created. Instead, cells become intermixed (Figure S6B). Analysis of the mixed regions indicated there was a significant increase in loser cell apoptosis when compared to the apoptosis of winner or lose cells lying outside the mixed region (Figure S6C-D). This suggests that loser cells are eliminated by short range signals.

© 2023. Published by The Company of Biologists under the terms of the Creative Commons Attribution License (https://creativecommons.org/licenses/by/4.0/).

Finally, as indicated above we have changed the ratio of wild-type to mutant cells and found that the higher the ratio of mutant cells the more rapid is the elimination of loser cells. We have not changed the ratio of *p53*$^{-/-}$ cells versus BCL2-overexpressing cells, as we do not think this would turn the BCL2-overexpressing cells into losers, given that the over-expression of the anti-apoptotic BCL2 will protect them from apoptotic elimination.

Minor comments:
1. Lines 77-79. Please check whether “8 x 10^5 cells” and “4 x 10^5 cells” are typos of “0.8 x 10^5 cells” and “0.4 x 10^5 cells”, respectively.

This typo has been corrected.

2. Line 130. “(1)” may be better described by “Eq. (1)” because the equation is referred to as “Eq. (1)” in Line 201.

This typo has been corrected.

3. Lines 231-232. The description “cells grown on the outside layer grew slower than those cultured on the inside” does not match with the data of Figure 4G. Please check this conclusion drawn from the data.

This typo has been corrected.

4. Lines 301-305. Please cite the authors’ previous paper(s) for Bowling et al., 2018.

This typo has been corrected.

Reviewer #2
Do p53-/- cells differentiate at the same rate as WT cells (Fig1) In fig.1 the authors observe that cell competition is most apparent upon culture in N2B27 and relatively negligible in pluripotency conditions. Line 98, the authors claim that “this behaviour was not due to the inability of p53 mutant cells to initiate differentiation”. This is an important point. When we compare Fig1 C and E, we observe that p53-/- cells proliferate faster during differentiation than during pluripotency whereas WT cells keep the same proliferation rate in both conditions in separate cultures. In Fig 1F we also see that Nanog in p53-/- is not downregulated as much as in WT cells and consistently, FGF5 is lower in p53-/- cells than in WT cells. These data indicate that p53-/- cells respond differently to the differentiation medium compared to WT cells. Therefore, there is a possibility that N2B27 induces a more heterogeneous differentiation in p53-/- cells or that p53-/- cells remain stuck in an intermediate differentiation stage that is more proliferative. If this is the case, one could argue that the competition behaviour inferred throughout the article is not a direct consequence of p53 sensing but instead an indirect effect of p53 KO promoting “winner” cell subpopulations in the culture.

To address this, the authors could stain for a few markers such as Oct4, SOX1, ECAD and NCAD and assess how efficient or homogeneous p53-/- differentiation is compared to the WT situation.

It has been shown by the Torres group that during cell competition, pluripotent cells eliminate those cells that initiate differentiation (Diaz-Diaz et al., Developmental Cell 2017). Therefore, it is conceivable that p53 mutant cells eliminate wild-type cells because they are more pluripotent than wild-type cells. To test this possibility, we have done two things. First, we have analysed, OCT4, SOX1, E-CADHERIN and N-CADHERIN expression in wild-type and p53 mutant cells after culture for 3 days in N2B27. We found that both cell types show similar levels of OCT4, E-CADHERIN and N-CADHERIN expression, and that *p53*$^{-/-}$ cells show slightly higher levels of SOX1 expression than wild-type cells (Fig. S1A-D). Second, we performed transcriptional profiling by RNAseq at the same time-point for both types of cells cultured separately. We found that when *p53* mutant cells were compared to wild-type cells, the most downregulated pathway was “p53 signalling pathway”, but the second one was “Signalling pathways regulating pluripotency in stem

© 2023. Published by The Company of Biologists under the terms of the Creative Commons Attribution License (https://creativecommons.org/licenses/by/4.0/).

cells" (Fig. S2E and Supplementary Table 1). This suggests a more rapid exit of pluripotency in *p53* mutant cells. Together, these data allow to exclude the possibility that *p53* mutant cells eliminate wild-type cells because they are more pluripotent. This data is discussed in lines 87-94 of the text.

Is WT cell elimination contact dependent (Fig4F-I). The "fences" experiment is very elegant but I think that some clarification could be added and that a simple additional experiment could strengthen the data:
1) I am surprised to see that the cells are growing faster when grown in the inner region, could the authors comment on the possible reasons for this observation?

This is an interesting point. Our assumption is that the density is lower in the inner region of the circular fences assay, and this facilitates faster growth. However, we have not tested this possibility as it is out of the scope what we could do for the revision of the paper.

2) The description of the experiment in the text is clear but I think that at least a bright field image of the culture would be helpful to illustrate the experimental setup and show the actual distance that separate the two population during the experiment.

Unfortunately, we do not have pictures at the time we performed this experiment, so do not have these images available. However, we hope that the images provided in Figure S7 using the alternative fences assay provide the perspective required by the reviewer.

3) To strengthen the idea that WT cell elimination requires short range signalling or cell contact, the authors could let the cells grow a sufficient amount of time in order for the 2 populations to reach one another and then quantify the level of cleaved caspase 8 at the front of encounter versus away from that front. Ideally this would be done in a different "fence format", for example using a removable 3-well insert as those provided by Ibidi for wound healing assays. WT cells would be seeded on wells 1 and 2 and p53-/- cells on well 3. Comparing the WT/WT interface vs the WT/p53-/- interface could provide strong evidence that contact is required for WT cell elimination by p53-/- cells.

To test this possibility, we have used an alternative fence assay from Ibidi, where the two wells are separated by a silicone insert that creates a defined cell free gap. Using this system winner and loser cells come together, but no straight boundary in created. Instead, cells become intermixed (Figure S6B). Analysis of the mixed regions indicated there was a significant increase in loser cell apoptosis when compared to the apoptosis of winner or lose cells lying outside the mixed region (Figure S6C-D). This suggests that loser cells are eliminated by short range signals.

Neighbourhood analysis (Fig5). The conclusion from the neighbourhood analysis shown in Fig 5A-D is not as intuitive as it is presented by the authors Line 269 "These results are consistent with cell neighbourhood having a role in the outcome of competition" For example, could these data be explained by differential growth rate only? Given that the net growth of p53-/- cells is much higher than WT cells in co-culture, we could reasonably expect that WT cells become progressively surrounded by p53-/- cells as p53-/- cells become the dominant population in the culture and this, regardless of whether neighbourhood plays a role or not (same for Bcl2 without Dox). Note that the Bcl2 rescue on the neighbourhood profile is interesting but Bcl2 also rescues the growth rate of the population. Conversely, if we follow the author's reasoning and assume that WT cells die when surrounded by p53-/- cells, shouldn't we expect surviving cells having mostly WT neighbours in the end (in contrast to what we see in the data)? I think it is not easy to mentally disentangle the contribution of these respective "forces" that dictate the neighbourhood profile. To address this, perhaps the authors could build on their existing mathematical model to predict the evolution of neighbourhood profiles in different scenarios and compare the results to their experimental data. If this is not feasible at this point (I appreciate the model does not currently take into consideration any spatial element), an alternative idea could be to stain for cleaved Caspase 8 at day3 or 4 and show that Caspase 8 negative WT cells tend to be surrounded by p53-/- cells whereas Caspase8 positive WT cells have a higher number of p53-/-cells.

As discussed above, we believe that the 2 well fence assay provides further support for the importance of cell neighbourhood for the elimination of wild-type cells as p53-/- cells. The fact that apoptosis significantly increases at the interface between wild-type and mutant cells

© 2023. Published by The Company of Biologists under the terms of the Creative Commons Attribution License (https://creativecommons.org/licenses/by/4.0/).

strengthens the case for the importance of local interactions governing the outcome of cell competition.

Minor comments:

* Line 88: I think a diagram explaining the strategy and the validation of the p53-/- cell line should be included in the manuscript (as a supplementary figure).

We have included the western blot showing complete absence of p53 protein in our mutant lines (Figure 1A) and indicated in the text the position of the mutation in the p53 gene (line 424-425 of the text).

* Line 145 One of the symbol in the equation is not defined.

We believe that all the symbols in equation 1 are defined. The symbols are the number of cells of each type N and P, the symbols for the intrinsic rates for each cell type $\rho_{!}$ with i = {W, K} i.e. $\rho^{\cdot\cdot}$ and $\rho_{\#}$; and the competition strengths $k_{\$}$ with j = {WW, WP, PW, P. We have highlighted the definitions in the paragraph above the equation containing these definitions.

* Line 145 Fig3A-B should be Fig 2.A-B

This typo has been corrected.

* Line 219 Eq (3) is not indicated in the Materials and Methods

The equations is defined in the Material and Methods section in the subsection: "Resource competition model".

* Fig. 4B For consistency, it would be good if the y axis could show "d0d4 ratio" as in other bar plots.

We have now replaced this graph.

* Line 234 – 237: "the fact that the number of cells in the culture dish can double without increasing the degree of wild-type apoptosis, also suggests that this elimination is not due to mechanical stress, as has been shown to be the case for MDCK cells with increased p53 expression" Here I assume that the assumption is that the cells are becoming more packed, hence the idea of mechanical stress. This could be more explicit, please clarify.

As suggested, in line 236-237 of the text, we now clarify that "the fact that the number of cells in the culture dish can double without increasing the degree of wild-type apoptosis, indicating that wild-type cells can become more packed without this increasing their apoptosis, also suggests that this elimination is not due to mechanical stress, as has been shown to be the case for MDCK cells with increased p53 expression".

Fig 4G is a bit misleading, at first it looks like the cells in the inner region proliferate slower than the cells in the outer region, while the opposite is true (based on Fig4. H). The graph could perhaps be represented with log scale for cell number or as time versus relative number of cells (cell number / initial number)

We agree that these growth curves are confusing. We have removed these graphs, as the final/initial cell numbers plotted in the new Fig. 4G-H (old Fig. 4H-I) provide the information required to understand the outcome of the experiment.

Fig. 5 – It can take some time to understand the histograms shown in Fig 5. It would be useful to add a label for the x axis. Maybe "% of WT cells". I did not understand why a + and – sign was shown in the graduation of the x axis. The y axis label is also confusing (removing the % sign could help).

We have now indicated that the X axis represents the % of WT cells with the given number of neighbours.

© 2023. Published by The Company of Biologists under the terms of the Creative Commons Attribution License (https://creativecommons.org/licenses/by/4.0/).

Fig. 5 E The results of the neighbour analysis at low density are clearly in favour of the author's thesis but to be even more convincing, I think the authors should show a comparison of the values of the growth rates shown in S5 A and B (to show that indeed, WT cells at low confluency are proliferating at their normal rate).

We have now provided the growth rates of wild-type and p53-/- cells between days 1 and 2, 2 and 3, as well as between days 3 and 4 for representative experiments. This shows as expected, that that wild-type and mutant cells grow faster at lower densities. This data is presented in Supplementary Table 1.

Reviewer #3:

1. Controls for CRISPR knockout: There are several essential controls missing. Generation/Use of the p53 CRISPR knock-out line is inadequately described. Are the "wild-type" cells isogenic controls? The methods and/or the text should clearly state what they are considering WT cells for these experiments. I also have concerns about off-target effects. There are no controls included to rule this out. At least some data with a second knock-out line should be shown before publication (and/or a rescue experiment would also be nice). Otherwise how do we know anything they show is specific for P53?

We are sorry for this omission. We have indicated in lines 442-443 of the text that the H2B-tdTomato ESCs (kind gift of Prof. Jenny Nichols, University of Edinburgh) were considered wild-type for the purpose of the described cell competition experiments. We also omitted to mention that all key experiments were repeated with a second *p53*$^{-/-}$ clone that showed the same results. These include the competition assays in pluripotency and differentiation conditions, as well as the analysis of differentiation markers shown in Figure 1. We have now included in Fig. 1A a western blot showing loss of P53 protein expression in both mutant clones and indicated in the methods that key experiments were done with two clones (lines 430-431).

2. Ruling out alternative models: It is very interesting that robust induction of cell competition only happens once cells exit from pluripotency. This also fits nicely with the timing of cell elimination events that the same group observes in vivo in the post-implantation mouse embryo. How ES cells are able to suppress cell competition in one state, but not another is a fascinating question. However, the relationship between differentiation and competition is not satisfactorily explored. While I fully appreciate that solving the potential role of P53 in transcriptional rewiring during the onset of differentiation is beyond the scope of this paper, I think the conclusions the authors make against this possibility are too strong. Is it not possible that subtle, heterogeneous differences in differentiation dynamics may also contribute to competitive outcomes? Simply exploring this with qPCR is not sufficient to rule this out. Moreover, although the data in Figure 1F shows similar trends between WT and P53 mutant cells, the magnitude is very different and the kinetics are also likely to be quite different (but this isn't explored). Ideally, more data should be added to address this, and at minimum, the conclusions should be softened. As discussed Oct4, SOX1, ECAD and NCAD in point 3 below, subtle differences in differentiation dynamics could potentially be another contributing factor to explain why the caspase stainings/inhibitor data doesn't seem to completely account for the overall growth disadvantage of WT cells.

As discussed in our response to reviewer 2, it has been shown by the Torres group that during cell competition, pluripotent cells eliminate those cells that initiate differentiation (Diaz-Diaz et al., Developmental Cell 2017). Therefore, it is conceivable that p53 mutant cells eliminate wild- type cells because they are more pluripotent than wild-type cells. To test this possibility, we have done two things. First, we have analysed, OCT4, SOX1, E-CADHERIN and N- CADHERIN expression in wild-type and p53 mutant cells after culture for 3 days in N2B27. We found that both cell types show similar levels of OCT4, E-CADHERIN and N-CADHERIN expression, and that *p53*$^{-/-}$ cells show slightly higher levels of SOX1 expression than wild-type cells (Fig. S1A-D). Second, we performed transcriptional profiling by RNAseq at the same time-point for both types of cells cultured separately. We found that when *p53* mutant cells were compared to wild-type cells, the most downregulated pathway was "p53 signalling pathway", but the second one was "Signalling pathways regulating pluripotency in stem cells" (Fig. S2E and Supplementary Table 1). This

© 2023. Published by The Company of Biologists under the terms of the Creative Commons Attribution License (https://creativecommons.org/licenses/by/4.0/).

suggests a more rapid exit of pluripotency in *p53* mutant cells. Together, these data allow to exclude the possibility that *p53* mutant cells eliminate wild-type cells because they are more pluripotent. This data is discussed in lines 87- 94 of the text.

3. Apoptosis data is not convincing: Although the Bcl2-induction experiments are very nice, the Caspase inhibitor data in Figure S2, together with the Cas-3/8 quantification shown in Figure 1H and Figure S2 doesn't sufficiently support the conclusion apoptosis is sufficient to explain the competitive loss of WT cells. The authors concede in the text that there might be something else going on but do not satisfactorily investigate these possibilities. As they mention, there may be caspase-independent death happening. There also may be live cell extrusion events without any caspase activation. Both of these possibilities may perhaps be addressed with live imaging experiments. Cell death is notoriously challenging to capture in IF – Casp3+ dying cells may be washed away and/or the time window of Casp3+ may be missed. Live imaging would allow them to get around these challenges and enable quantification of a wider diversity elimination events rather than just caspase-dependent apoptosis-per se. I am also wondering about senescence? Is it possible that WT cells instead enter a senescent state? Although perhaps less likely to be rescued by Bcl2 over-expression, wt cells becoming increasingly senescent would be somewhat in line with what has been observed in the hematopoietic system by the Medzhitov lab. This could be addressed with a quick IF staining.

The mode of elimination of loser cells is an important issue. In the new Figure S7, where wild- type and p53 mutant cells come together in a more controlled fashion, we find significantly increased levels of apoptosis in the loser cells that have become mixed with the mutant cells, when compared to those in non-mixed areas. This, together with our observation that constitutive-expression of the anti-apoptotic factor BCL-2 completely rescues loser cell elimination, indicates that apoptosis is the primary mode of elimination of wild-type cells by p53 mutant cells. It is worth highlighting, that inhibitor experiments are never completely clean; therefore, it is not completely surprising we didn't see a complete rescue using caspase inhibitors. Given this, we have not attempted to unravel if there could be other contributing modes of loser cell elimination, as the pathways involved in this mode of elimination are not the focus of the current manuscript.

4. Ruling out Mechanical Competition: I don't think the authors can sufficiently rule this out and I don't fully understand their rationale for doing so based solely on the data from the perfusion experiments. Perhaps one source of my confusion is that there is plenty of quantification of cell number, but no discussion or quantification of confluence or of cell density. In fact, the neighbourhood data in Figure 5 also suggests that cell density could play a role. This should be explored.

To address this point, we have done two things. First, we have calculated the wild-type rate of elimination when co-cultured with p53 mutant cells in a 1:1 ratio when the following cell densities are plated: $0.16x10^5$ cells, 0.16x105, $0.8x10^5$ cells, $2x10^5$ cells and $10x10^5$ cells. We find that between days 3 and 4 this rate of elimination is highest when $2x10^5$ cells are plated, but then is 60% lower when five time more cells ($10x10^5$ cells) are platted. This data is presented in Supplementary Table 2. Similarly, when $0.4x10^5$, $0.8x10^5$, $1.6x10^5$ and $3.2x10^5$ cells are seeded, normalised Caspase 8 levels in wild-type cells peak at the $1.6x10^5$ seeding density and then decrease when $3.2x10^5$ cells are seeded. This data is presented in Figure S6. Together, these results suggest that there is not a direct correlation between cell density and loser cell elimination and suggests that mechanical competition is not the main driver for this elimination. This is discussed in lines 239-246.

5. Mathematical Model Confusion: this reviewer is not a mathematician but I am a biologist with great appreciation for cross-disciplinary approaches. I tried my best to make sense of the data from the model but I had a pretty hard time ascertainin gthe parameters and the parameter space explored. I understand the value of the conclusions but how we got there is a little bit opaque from my perspective and I suspect I would not be alone on this amongst the Development readership. In more concrete terms what is entailed in the competition "strength" parameter k? The manuscript cites a prior study of bacterial population dynamics where a similar model was

© 2023. Published by The Company of Biologists under the terms of the Creative Commons Attribution License (https://creativecommons.org/licenses/by/4.0/).

previously used, but it would help to more explicitly explain this if possible. I also struggled with Figure 2D which is of course absolutely critical to understand the parameters that actually fit the experimental data and (I think?) encapsulates the results that are the key utility of the mathematical model. If the authors could come up with a complimentary way to convey which part of the parameter space the experimental data occupies, this would be very helpful. I suspect that the data shown in Figure S4 partly addresses this comment but I am not at all equipped to understand what is shown in that Figure – this could also be much better explained than it currently is. Perhaps another schematic (similar to Figure 2A, B which are fantastic) would also be useful? Or could the inferred parameters somehow be indicated on that pre-existing schematic? As I read on, the data shown in Figure 3I was immensely helpful to clarify for me how the model was working – ie. examples of how changing the parameters can fit different experimental conditions. In my view, it may be worth considering restructuring the paper somewhat so that this data can go together with the initial description of the model. I think the fact that until then we only see modelling data from one set of parameter values makes it a bit challenging to appreciate and understand the range of possibilities and the potential predictive power of the model.

We are very grateful for this insightful feedback on the presentation of the mathematical model. We agree with the referee on the relevance to design towards the wide readership of Development. In order to do so we have clarified the meaning of the interaction strength parameters when the equations of the model are introduced: “In particular, parameters k_j control competition due to crowded cell populations and accommodate for the possibility of different apoptotic rates for each cell type in homotypic (k_WW and k_PP) and heterotypic (k_WP and k_PW) environments”. In addition, we have made more explicit how to interpret the posterior distributions from the analysis. As the referee pointed out this information is in Supplementary figure S4 and condensed in figure 2D. These probability distributions contain all the power for hypothesis testing. They return all the parameter sets compatible with the data and allow us to quantify if a certain parameter is larger than another (e.g. k_ww > k_wp?) by looking at the fraction of the posterior distribution in which that inequality is satisfied. This is now explained in the text making emphasis in the range of hypothesis compatible with the parameter exploration:
“The initial parameter space explored (the prior distribution) included parameter regions equally compatible with different hypothesis. These distributions will be constrained by the experimental data resulting in parameter distributions (the posterior distribution) that can be used to perform hypothesis testing (e.g. we can study if intrinsic proliferation rates differ from each other by analyzing the fraction of the posterior distribution in which ρ_P>ρ_W)”.

Similarly, we have added another sentence closer to the result section regarding how to interpret the pairwise posterior distributions: “Most importantly, the resulting distributions allow us to compare different hypotheses through the pairwise relationships between parameters. This can be done by evaluating the extent of parameter regions that satisfy inequalities between different parameters. (Fig. 2D and S4).”

Finally we have redone Figure 2D using similar diagrams than 2A-B to make the interpretation of the inference more visual.

Minor Points:
-figure call-outs are not always correct,

We have done this correction.

-I assume the TdTomato cells in Fig 5 are the WT cells but this isn’t explicitly labelled anywhere on the figure or in the figure legend/text (unless I missed it).

This has now been indicated in the Figure and in lines 442-443 of the text.

Resubmission

© 2023. Published by The Company of Biologists under the terms of the Creative Commons Attribution License (https://creativecommons.org/licenses/by/4.0/).

First decision letter

MS ID#: DEVELOP/2023/202503

MS TITLE: Mutation of p53 increases the competitive ability of pluripotent stem cells.

AUTHORS: Salvador Perez Montero, Pranab Kumar Paul, Aida Di Gregorio, Sarah Bowling, Solomon Shepherd, Nadia J Fernandes, Ana Lima, Ruben Perez-Carrasco, and Tristan A Rodriguez

I have now received all the referees reports on the above manuscript, and have reached a decision. The referees' comments are appended below, or you can access them online: please go to BenchPress and click on the 'Manuscripts with Decisions' queue in the Author Area.

The overall evaluation is positive and we would like to publish a revised manuscript in Development, provided that the referees' critique can be satisfactorily addressed. Please attend to all of the reviewers' comments in your revised manuscript and detail them in your point-by-point response. If you do not agree with any of their criticisms or suggestions explain clearly why this is so.

Reviewer 1

Advance summary and potential significance to field

P53 is suggested to be involved in the elimination of loser cells in several cell competition systems. This paper describes a novel finding that p53-mutant cells behave as super-competitors, eliminating wild-type cells from cell competition during the onset of differentiation of ES cells. The authors also demonstrated through a combination of mathematical modeling and cell culture experiments that the elimination of wild-type cells by p53 mutant cells is mediated by short-range interactions, not by the deprivation of nutrients or mechanical stresses.

Finally the authors showed that changes in the local cell neighborhood correlate with changes in cell number and concluded that the relative number of neighboring cells with different competitive abilities influences the replacement of wild-type cells.

These findings are important for the field of cell competition and developmental biology because they introduce a new distinct mode of cell competition during ES cell differentiation and reveal a novel role of p53 in cell competition.

Comments for the author

The authors have adequately addressed all the comments provided by the reviewers.
After the incorporation of the minor comments, the paper is now suitable for publication.

Minor comments:
1. Some errors appear to be present. Kindly review.
    - Line 139: "i = (Abbadie et al., 1993)"; the explanation of “i” is missing.
    - Incorrect citation for some figures: line 149 ("Fig. 2A top") and line 228 ("Fig. S5"). Please rectify.

Reviewer 2

Advance summary and potential significance to field

I have read through the revised manuscript in details and I am pleased with the authors' responses to my comments. I have no further comment and I am recommending the work by Montero and colleagues for publication in Development. I think this will be an article of great interest to the community.

© 2023. Published by The Company of Biologists under the terms of the Creative Commons Attribution License (https://creativecommons.org/licenses/by/4.0/).

Comments for the author

-

Reviewer 3

Advance summary and potential significance to field

The manuscript from the Tristan Rodriguez group follows on a growing body of work including their own, studying mechanisms of cell competition in mouse ES cells. In particular, they seek to understand the mechanisms by which p53 mutant cells out-compete wild-type cells – a phenomenon previously observed in vitro and in vivo in mouse embryos and interspecies chimeras. Working with mouse ES cells in vitro they use a combination of mathematical modelling and cell-based assays to characterize the cellular and molecular mechanisms that underlies this observation. Their overall conclusion is that short-range signaling (ie. Direct cell-cell contact) between wt and P53 mutant cells induces wt cell apoptosis, and that this does not occur by virtue of any competition for nutrients or space. A majority of the cell competition scenarios described in mammals seem to converge on P53, and thus the insights this manuscript provides of the mechanisms by which P53 mutant cells super-compete could be generalizable across many other biological contexts. As such, the readership of Development will find this paper a noteworthy contribution to our understanding of growth control in developing mammalian tissues.

Comments for the author

The manuscript is much improved and very nice to read. All of the points raised have been addressed thoughtfully in the revised version. The efforts to improve the description of the math are particularly appreciated! In my view, it is now suitable for publication in Development, with a few minor points that should be addressed as follows:
1. Interpretation of Figure 1B-C: it is a bit confusing to me the way this data is discussed in the text because there actually is a significant difference between WT and P53 growth in co-culture in pluripotent conditions. I understand its much less than what is observed in differentiation conditions (Figure 1D-E) but I nevertheless think more clearly acknowledging the small but significant difference in growth on line 79 of the text would be more accurate.
2. Figure 3: I would be curious to know more about the role of the timing of Dox administration to the data shown in Figure 3H-I. From what I understand most of the BCL2 data in Figure 3 is from Dox administration at Day 0 but the data in 3I is from administration at Day 2. I suppose figure 3H shows that the chosen parameters of the model recapitulate quite well the experimental results no matter what the timing of Dox administration is, but it might be helpful, if space allows, to add an additional sentence of text to clarify about the issue of timing.
3. Figure 4: stats should be added to Figure 4g 4. Figure 5: the Y-axis labels on the neighbourhood plots are a bit confusing. Doesn't this reflect an absolute number of cell neighbours? But the label says "number of neighbours (%)" and this is confusing. If I understand correctly, reviewer 2 may also have raised this point last time. I believe the % symbol on the Y-axis labels should be removed.
5. Figure S3: why are no images shown for Cleaved caspase 3? If possible it would be nice to also include these here.
6. Figure S3C: a label indicating that RFP cells are WT would be helpful. I also am not really sure the images selected show what is claimed in the text and quantified in Figure 1I. To my eye it appears in the co-culture image there are quite a lot of RFP-negative Caspase 8+ cells. I would therefore suggest inserting a different image or adjusting the presentation of this data so that the images and quantifications match more clearly.
7. Figure S3D: the Y-axis label should be clarified to include fold change in cell number at d4. Fold change by itself is a bit confusing.
8. Figure S7: In my opinion, this is critical, very convincing data that should be included in a main figure if possible (Figure 4 for example).
9. Line 167 – it says 24 different plating conditions were attempted. But I only count 22… (16 in Fig S4 and 6 in Figure 2C)
10. Line 241: typo – depravation should be deprivation.

© 2023. Published by The Company of Biologists under the terms of the Creative Commons Attribution License (https://creativecommons.org/licenses/by/4.0/).

First revision

Author response to reviewers' comments

Response to the Reviewers
We are grateful for the supportive comments of the reviewers. We detail below our responses to the specific points that they raised and indicate what changes to the manuscript have been performed.

Reviewer #1
Minor points:
Line 139: "i = (Abbadie et al., 1993)"; the explanation of "i" is missing.
Incorrect citation for some figures: line 149 ("Fig. 2A top") and line 228 ("Fig. S5"). Please rectify.
We thank the referee for spotting these typos, they have been fixed in the new version of the manuscript.

Reviewer #3
Minor points:
1. Interpretation of Figure 1B-C: it is a bit confusing to me the way this data is discussed in the text because there actually is a significant difference between WT and P53 growth in co-culture in pluripotent conditions. I understand its much less than what is observed in differentiation conditions (Figure 1D-E) but I nevertheless think more clearly acknowledging the small but significant difference in growth on line 79 of the text would be more accurate.
We thank the reviewer for pointing this out. We now indicate this by saying the following: "We found that in pluripotency culture conditions, p53 mutant cells grew at a similar rate to wild-type cells in separate culture and displayed a small growth advantage in the co-culture condition (Fig. 1B-C)".

2. Figure 3: I would be curious to know more about the role of the timing of Dox administration to the data shown in Figure 3H-I. From what I understand most of the BCL2 data in Figure 3 is from Dox administration at Day 0 but the data in 3I is from administration at Day 2. I suppose figure 3H shows that the chosen parameters of the model recapitulate quite well the experimental results no matter what the timing of Dox administration is, but it might be helpful, if space allows, to add an additional sentence of text to clarify about the issue of timing.
In lines 201-205 of the text, we now clarify this point by saying "Importantly, the longer the cells were treated with doxycycline, the better the rescue is: co-cultures treated from day 0 show a complete rescue, adding doxycycline from day 2 reduces the level of rescue and adding it from day 3 reduces the rescue further (Fig. 3H)".

3. Figure 4: stats should be added to Figure 4g
These have now been added.

4. Figure 5: the Y-axis labels on the neighbourhood plots are a bit confusing. Doesn't this reflect an absolute number of cell neighbours? But the label says "number of neighbours (%)" and this is confusing. If I understand correctly, reviewer 2 may also have raised this point last time. I believe the % symbol on the Y-axis labels should be removed.
We thank the reviewer for spotting this, as indeed it was confusing. We have now removed the % sign from the Figure 5.

5.Figure S3: why are no images shown for Cleaved caspase 3? If possible it would be nice to also include these here.
We apologise for the lack of clarity. The percentage of cleaved Caspase 3 positive cells was determined by FACS. This had been indicated in the methods section and we now also indicate it in the figure legend.

© 2023. Published by The Company of Biologists under the terms of the Creative Commons Attribution License (https://creativecommons.org/licenses/by/4.0/).

6. Figure S3C: a label indicating that RFP cells are WT would be helpful. I also am not really sure the images selected show what is claimed in the text and quantified in Figure 1I. To my eye it appears in the co-culture image there are quite a lot of RFP-negative Caspase 8+ cells. I would therefore suggest inserting a different image or adjusting the presentation of this data so that the images and quantifications match more clearly.
As suggested, we have added the tdTomato label and changed the selected images.

7.Figure S3D: the Y-axis label should be clarified to include fold change in cell number at d4. Fold change by itself is a bit confusing.
We have not changed this, as we have used this Y-axis label in all figures showing this type of data. The figure legends contain the necessary explanatory details.

8.Figure S7: In my opinion, this is critical, very convincing data that should be included in a main figure if possible (Figure 4 for example).
We have now made this figure into new Figure 5.

9. Line 167 – it says 24 different plating conditions were attempted. But I only count 22… (16 in Fig S4 and 6 in Figure 2C)

Thank you for pointing out these potential confusions. Each condition corresponds to an individual experiment. In Fig. S4, there are 8 co-culture experiments, 8 experiments of single cultures of WT, and 8 experiments of single cultures of p53-/-. In total, this amounts to 24 experimental conditions. We have clarified that this number refers only to the new conditions shown in Fig S4 by explicitly stating that they are 'additional conditions.

10. Line 241: typo – depravation should be deprivation.
This has been corrected.

Second decision letter

MS ID#: DEVELOP/2023/202503

MS TITLE: Mutation of p53 increases the competitive ability of pluripotent stem cells.

AUTHORS: Salvador Perez Montero, Pranab Kumar Paul, Aida Di Gregorio, Sarah Bowling, Solomon Shepherd, Nadia J Fernandes, Ana Lima, Ruben Perez-Carrasco, and Tristan A Rodriguez
ARTICLE TYPE: Research Article

I am satisfied with the revision of the manuscript. This manuscript has been accepted for publication in Development, pending our standard ethics checks.

© 2023. Published by The Company of Biologists under the terms of the Creative Commons Attribution License (https://creativecommons.org/licenses/by/4.0/).
